# Affimer Tagged Cubosomes: Targeting of Carcinoembryonic Antigen Expressing Colorectal Cancer Cells Using *In Vitro* and *In Vivo* Models

**DOI:** 10.1021/acsami.1c21655

**Published:** 2022-02-23

**Authors:** Arindam Pramanik, Zexi Xu, Shazana H. Shamsuddin, Yazan S. Khaled, Nicola Ingram, Thomas Maisey, Darren Tomlinson, P. Louise Coletta, David Jayne, Thomas A. Hughes, Arwen I. I. Tyler, Paul A. Millner

**Affiliations:** † School of Biomedical Sciences, University of Leeds, Leeds LS2 9JT, United Kingdom; ‡ School of Medicine, University of Leeds, Leeds LS9 7TF, United Kingdom; § School of Food Science and Nutrition, University of Leeds, Leeds LS2 9JT, United Kingdom; ∥ School of Chemistry and Astbury Centre for Structural Molecular Biology, University of Leeds, Leeds LS2 9JT, United Kingdom; ⊥ Department of Pathology, School of Medical Sciences, Universiti Sains Malaysia, George Town 16150, Malaysia; # Leeds Institute of Medical Research, St James’s University Hospital, Leeds LS9 7TF, United Kingdom; % Biomedical Health Research Centre, BioScreening Technology Group, University of Leeds, Leeds LS2 9JT, United Kingdom

**Keywords:** Affimers, cubosomes, lipids, lyotropic liquid crystalline nanoparticles, cancer, active targeting

## Abstract

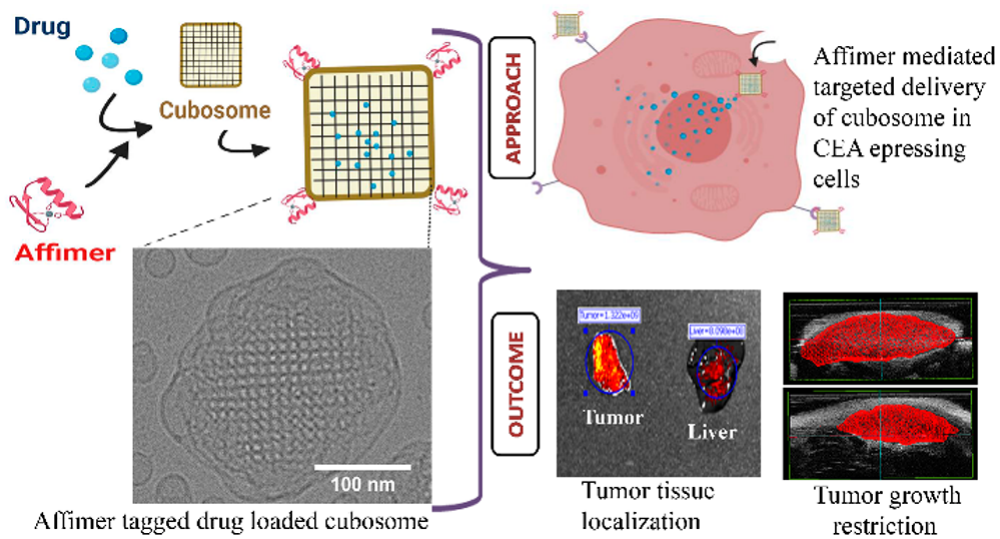

Nanomedicines, while having been approved for cancer therapy, present many challenges such as low stability, rapid clearance, and nonspecificity leading to off-target toxicity. Cubosomes are porous lyotropic liquid crystalline nanoparticles that have shown great premise as drug delivery vehicles; however, their behavior *in vivo* is largely underexplored, hindering clinical translation. Here, we have engineered cubosomes based on the space group *Im*3*m* that are loaded with copper acetylacetonate as a model drug, and their surfaces are functionalized for the first time with Affimer proteins via copper-free click chemistry to actively target overexpressed carcinoembryonic antigens on LS174T colorectal cancer cells. Unlike nontargeted cubosomes, Affimer tagged cubosomes showed preferential accumulation in cancer cells compared to normal cells not only *in vitro* (2D monolayer cell culture and 3D spheroid models) but also *in vivo* in colorectal cancer mouse xenografts, while exhibiting low nonspecific absorption and toxicity in other vital organs. Cancerous spheroids had maximum cell death compared to noncancerous cells upon targeted delivery. Xenografts subjected to targeted drug-loaded cubosomes showed a 5–7-fold higher drug accumulation in the tumor tissue compared to the liver, kidneys, and other vital organs, a significant decrease in tumor growth, and an increased survival rate compared to the nontargeted group. This work encompasses the first thorough preclinical investigation of Affimer targeted cubosomes as a cancer therapeutic.

## Introduction

**Figure 1:**
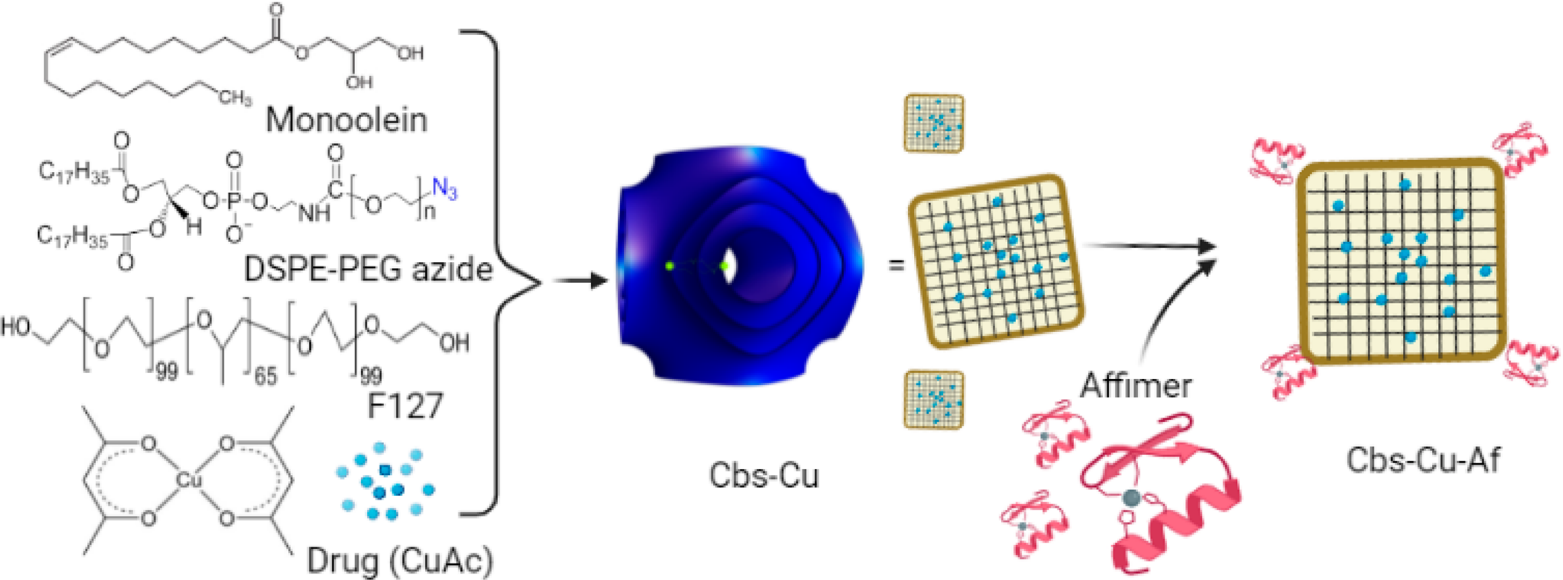
Monoolein-based dispersions of the primitive inverse bicontinuous cubic phase (cubosomes, shown in blue), which is based on space group *Im*3*m*, were engineered to encapsulate the model organometallic drug copper acetylacetonate (CuAc). The nanoparticles were stabilized by Pluronic F127 and DSPE-PEG2000-azide. DSPE-PEG2000-azide in the outer corona allowed conjugation of Affimer proteins, engineered to have a DCBO functional group, to the cubosome via copper-free click chemistry to target overexpressed carcinoembryonic antigens on colorectal cancer cells.

Nanomedicine is an emerging field that has shown great potential in providing state-of-the-art diagnosis and treatment of many diseases and a plethora of nanoparticle formulations have been developed based on proteins, polymers, lipids, metals, or inorganic elements.^1^ An emerging class of lipid-based nanoparticles are dispersions of inverse lyotropic liquid crystalline phases. These have internal nanostructures that possess two- or three-dimensional periodicity, such as hexagonal or cubic symmetries, and are usually stabilized by a polymer corona. These lyotropic liquid crystalline lipid nanoparticles (LCNPs) offer several advantages such as structural versatility, porosity, improved stability, high encapsulation efficiency due to their high internal surface area, and biocompatibility due to mostly being made up of food-grade material.^2,3^ Cubosomes, a type of LCNPs, have attracted interest as delivery vectors for theranostic applications. They have an internal structure based on either the diamond, primitive, or gyroid bicontinuous cubic phases belonging to space groups *Pn*3*m*, *Im*3*m*, and *Ia*3*d*, respectively, and consist of two noncommunicating water channels divided by a single continuous lipid bilayer (Figure 1).^2,3^ Cubosomes have the potential to offer controlled release of encapsulated actives^4−6^ that can also be achieved via phase transitions in response to a stimulus such as pH^7,8^ as well as facilitated cellular uptake.^9,10^ Because of their amphiphilic nature, they can encapsulate hydrophilic and hydrophilic cargo^2^ including drugs, imaging agents,^11^ and biomolecular payloads such as proteins,^12^ DNA,^13^ or small interfering RNA.^14^ LCNPs have been reported to have superior performance and efficacy of the loaded cargo in a variety of disease sites and models.^3^ For example, cubosomes outperformed liposomes in siRNA delivery and transfection.^14^

While many anticancer drugs have been encapsulated into cubosomes and tested for efficacy in a number of different cell lines to mimic various disease models with promising results,^15−17^ these have mostly been based on passive targeting of nanoparticles, which often requires high drug loading that can lead to off-target toxicity. Cubosomes made entirely of polymers have also been recently synthesized,^18,19^ although to the best of our knowledge there have been no studies on the encapsulation of actives within them or their use in biomedical applications. While polymer nanoparticles offer advantages such as increased stability compared to their lipid counterparts, they also suffer from disadvantages such as low biocompatibility and increased cytotoxicity compared to their lipidic counterparts. A small number of studies have functionalized the outer corona of lipid-based cubosomes with molecules such as biotin,^20^ folate,^21^ and epidermal growth factor receptor antibody fragments,^22^ which showed high affinity and specificity to their target. Alcaraz et al. developed cubosomes that could undergo copper-free click chemistry that have the potential to target cell surfaces by metabolic labeling.^23^ Moreover, investigation of cubosome–cell interactions has been limited to 2D monolayer cultures. Recently Zhai et al. explored the interaction of paclitaxel-loaded cubosomes with 3D spheroid models of skin cancer cells, which provides a much more relevant *in vitro* model to mimic *in vivo* conditions, and found that cancer cells in the spheroids were more resistant to treatment compared to 2D models.^9^

Very few studies have focused on the cytotoxicity and, in particular, the biodistribution of cubosomes *in vivo*(3,9,11,22,24) and as all these studies have had different theranostic applications, used different administration routes, and had varied compositions of lipids and stabilizer, it is difficult to draw conclusions on the biological fate of cubosomes. For example, Biffi et al. showed that fluorescent monoolein cubosomes administered intravenously to the tail vein of healthy mice preferentially accumulated in the liver as monitored over time and up to 48 h postinjection.^25^ On the contrary, intraperitoneal injection of paclitaxel-loaded monoolein cubosomes to A431 skin cancer mouse xenografts showed preferential accumulation at tumor sites, monitored up to 24 h postinjection.^9^

In this work, we aim to develop active cancer-targeted cubosomes to colorectal cancer cells loaded with a model anticancer drug and investigate their efficacy both *in vivo* and *in vitro* and their efficacy and biodistribution *in vivo*—the first study to perform such a thorough preclinical investigation. The heterogeneity between individual colorectal cancers (CRC) and the lack of consistently overexpressed receptors that can be used as biomarkers limit targeted drug delivery.^26^ We have previously shown that the most suitable surface biomarker in CRC, in terms of both degree and frequency of overexpression, is a carcinoembryonic antigen (CEA).^27^ CEA has been used as a biomarker to image CRC *in vivo* by using fluorescent silica nanoparticles tagged with monoclonal antibodies (mAb).^28^ Bottlenecks associated with mAb-based drug conjugates, however, include the high cost of production, stability, and batch-to-batch variation, which limit their clinical development.^29,30^ Affimers are small proteins that are engineered to have similar binding and specificity as mAbs but offer advantages such as increased stability over a range of conditions (temperature, pH) and ease of production/scaleup, thereby ensuring consistency over batch-to-batch productions while maintaining specific target recognition.^31−34^ Affimers, identified from a phage display library, that have specificity toward CEA antigens have been developed^35^ and exhibit ease of surface functionalization on molecules of interest.^36^

Here we have developed monoolein (MO)-based cubosomes (Figure 1) that encapsulated 5 wt % (with respect to MO) of the model organometallic cancer drug copper acetylacetonate (CuAc). We were specifically interested in relatively simple copper compounds as anticancer agents as they have potential to provide novel and low-cost drugs that could be affordable in a global context.^37^ We have previously shown that CuAc has potent anticancer activity; however, because of its poor solubility and cytotoxicity, an encapsulation strategy is necessary.^38,39^ The CuAc-loaded cubosomes were targeted to CRC cells by using Affimers, attached on the cubosome’s surface via copper-free click chemistry. Cubosomes were characterized by using small-angle X-ray scattering (SAXS), cryogenic transmission electron microscopy (cryo-TEM), and dynamic light scattering (DLS). The therapeutic efficacy of the nanoformulation was studied *in vitro* in both CRC 2D monolayer cultures and 3D spheroids as well as tumor xenograft bearing mice and showed selectivity toward CEA expressing cells. Cancerous spheroids showed maximum cell death compared to noncancerous cells upon targeted delivery, and CRC xenografts showed a large decrease in tumor volume, no off-target toxicity, and increased survival rates. The localization of the cubosomes both *in vitro* and *in vivo* was also studied by using fluorescence tags and showed preferential uptake of the targeted cubosomes by cancerous CEA expressing cells.

## Results and Discussion

### Characterization of Clickable Cubosomes Tagged with Affimer Protein

We formulated and characterized monoolein (MO)-based cubosomes stabilized by Pluronic F127 and DSPE-PEG2000-azide and loaded with a model hydrophobic drug. DSPE-PEG2000-azide, apart from acting as a stabilizer, has the additional role of allowing surface functionalization of the cubosomes with any ligand with dibenzocyclooctyne (DBCO) groups via copper-free click chemistry. Appreciating that size might be an important consideration when designing nanocarriers,^40−42^ as larger particles (>200 nm) may potentially limit their ability to reach the tumor tissue whereas smaller particles (<20 nm) have low retention in the tumor and fast clearance *in vivo*,^43^ we explored different MO:F127:DSPE-PEG ratios and their effect on particle size (Table S1). Preliminary exploration of dispersion conditions found that dispersing the particles in an ice bath gave smaller particles sizes on average compared to dispersing at room temperature. Out of the compositions tested, MO:DPA:F127 88.79:4.67:6.54 (w/w) yielded the smallest *Z*-average diameter of 106 nm as well as the lowest polydispersity index (PDI) of 0.18, and hence this concentration of MO:DPA:F127 was taken forward for all subsequent experiments. It should be noted that the hydrodynamic diameter of the cubosomes as measured by dynamic light scattering (DLS) is not the same as their physical size. The mean size of the nanoparticles obtained from various techniques weighs the size distribution differently so, for example, DLS data will emphasize larger particles whereas cryo-TEM often excludes larger particles from the thin ice and hence highlights smaller particles in these polydisperse samples (see comparisons later). Complexes of platinum, ruthenium, titanium, and gallium have successfully entered clinical trials, leaving potential for other complexes to be researched as cancer therapeutics.^44^ We have used one such metal–organic complex of copper, copper acetylacetonate (CuAc), as a model hydrophobic drug in this study. This complex has been extensively studied in various cancer cells in our previous reports.^38,39^ In this study, we found that encapsulating 5% (w/w with respect to MO) of CuAc in cubosomes (Cbs) to be optimum, ensuring stable dispersions for up to 21 days (Table S2), and hence this loading was used in subsequent studies. The encapsulation of CuAc in the cubosome was confirmed by energy-dispersive X-ray spectroscopy (EDAX). As shown in Figures S1A and S1B, a distinct peak for Cu was noted as expected at 8 keV, which was not present in the analysis of cubosomes without CuAc. Inductively coupled plasma optical emission spectrometry (ICP-OES) using Cu as a reference material could further evaluate the encapsulation efficiency of CuAc in the cubosome (Table S2). Similar to Bazylińska et al.,^45^ where a high encapsulation efficiency was noted for a photosensitizer (Ce6) loaded cubosome, the encapsulation efficiency of 5 wt % CuAc (with respect to MO) in our study was found to be 82 ± 4.0% (Table S2). DBCO labeled Affimers were conjugated to the Cbs-Cu (CuAc loaded cubosome) via copper-free click chemistry. As shown by the FT-IR spectra (Figure S1C), a peak at 2127 cm^–1^ is observed for Cbs-Cu, which signifies the presence of an azide group. The peak disappears for Affimer tagged Cbs-Cu (Cbs-Cu-Af) due to the covalent bonding of the DBCO labeled Affimer to the azide group of DSPE-PEG in the cubosome. Affimer conjugation is further confirmed by using EDAX data (Figure S1B) which show the characteristic sulfur Kα and Kβ peaks (2.3–2.5 eV) arising from the tagged cubosomes that is due to cysteine present in the Affimer. These peaks are absent in the bare cubosomes (Figure S1A). The Cbs-Cu-Af cubosomes showed a prolonged and sustained release of CuAc from the nanoparticles which was up to 60% of its total encapsulation even after 48 h (Figure S1D).

**Figure 2:**
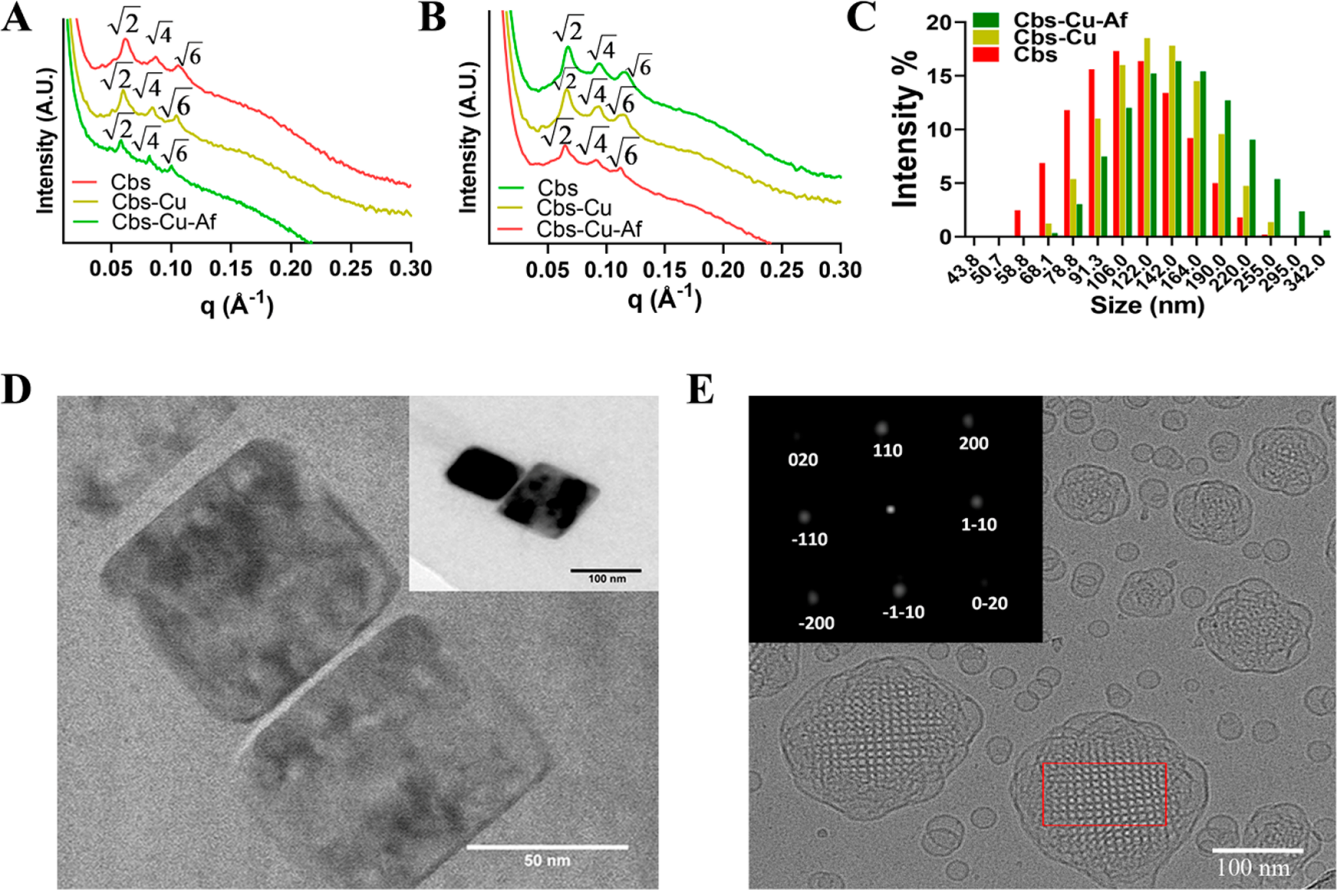
Cubosome characterization. SAXS patterns of Cbs, Cbs-Cu, and Cbs-Cu-Af at (A) 25 °C and (B) 37 °C. All SAXS patterns index to a primitive bicontinuous cubic phase belonging to space group *Im*3*m*. (C) DLS data showing the hydrodynamic diameter of Cbs, Cbs-Cu, and Cbs-Cu-Af with a *z*-average size of 106, 121, and 141 nm, respectively. (D) TEM image Cbs-Cu-Af and (E) representative cryo-TEM image of Cbs-Cu-Af. The corresponding intensity of the fast Fourier transform (FFT) applied to the cubosome (red box) is shown in the inset along with the assigned Miller indices which index to space group *Im*3*m*.

The internal nanostructure of the bare (Cbs), drug loaded (Cbs-Cu), and drug and Affimer tagged (Cbs-Cu-Af) cubosomes was studied by small-angle X-ray scattering (SAXS) at 25 and 37 °C (Figures 2A and 2B). The choice of 25 °C justified storage at room temperature and long-term stability, whereas 37 °C justified its stability at a physiological relevant temperature. All SAXS patterns show Bragg peaks in the ratio of √2:√4:√6 (which correspond to Miller indices (*hkl*) 110, 200, and 211) which index as a primitive bicontinuous cubic space belonging to space group *Im*3*m*. The lattice parameters of Cbs, Cbs-Cu, and Cbs-Cu-Af cubosomes at 25 °C were 144.9, 149.3, and 153.8 Å and were 133.3, 135.1, and 138.9 Å at 37 °C, respectively. MO is known to form a bicontinuous cubic phase of space group *Pn*3*m* in excess water at the temperature range explored in this study;^46,47^ however, when Pluronic F127 is used to stabilize dispersions of MO, the system transforms to an *Im*3*m* phase.^48^ Our results as well as the lattice parameters obtained here are consistent with previous studies on bare cubosomes.^9,23^ Encapsulated 5 wt % CuAc does not cause a phase transition but slightly increases the lattice parameter as the bulky metal complex decreases the magnitude of the monolayer spontaneous inverse curvature. Similarly, addition of DPA causes a further increase in the lattice parameter as it decreases the hydrocarbon chain splay resulting in less curved structures as was previously shown in phytantriol-based cubosomes.^23^ The *Z*-average sizes of Cbs, Cbs-Cu, and Cbs-Cu-Af cubosomes at 25 °C were 106, 121, and 141 nm and had a polydispersity index of 0.155, 0.159, and 0.086 respectively (Figure 2C and Figure S2). It should be noted that although DLS data on phytantriol:DPA cubosomes showed a bimodal distribution and a significantly larger average size and PDI,^23^ this was not the case for our MO-based cubosomes. The mean size of Cbs-Cu-Af cubosomes was also calculated by nanoparticle size analysis from cryo-TEM data and gave a mean size of 130 nm (Figure S3A). This is comparable to the value of 141 nm obtained by DLS. TEM nanoparticle size analysis of Cbs-Cu-Af gave a mean size of 66 nm (Figure S3B) although care should be taken when interpreting this number as TEM measurements of soft materials can lead to deformation and mass loss of the sample.

The shape and morphology of Cbs-Cu-Af cubosomes were visualized by transmission electron microscopy (TEM) (Figure 2D), which showed a neat cubical structure. Their internal nanostructure was further visualized by cryo-TEM (Figure 2E). Cryo-TEM images show ordered internal nanostructures which index to space group *Im*3*m* (Figure 2E, inset). Cryo-TEM images show *Im*3*m* cubosomes with a small number of vesicular structures which is known and caused due to a surplus of F127.^49^

### Carcinoembryonic Antigen Is a Suitable Marker for Colorectal Cancers

Carcinoembryonic antigen (CEA) has been reported as a marker on the surface of cancer cells including lung, breast, and pancreatic, yet predominantly its expression has been noted in colon and rectum cancers, as found from clinical samples.^50−52^ We have previously reported LoVo CRC cell lines having a relatively high expression of CEA.^35^ In this study, we show LS174T CRC cell lines exhibiting high CEA expression compared to noncancerous HEK-293 cells using CEA mAb tagged with the Alexa Fluor 488 secondary antibody (Figure S5A). This was further validated by using a Western blot from both LS174T and HEK-293 cell lysates (Figure S5B), where the expression of CEA was found to be 9-fold higher in the case of LS174T cells compared to HEK-293.

### CEA Affimers Successfully Target Cubosomes to CEA Expressing Colorectal Cancer Cells

**Figure 3:**
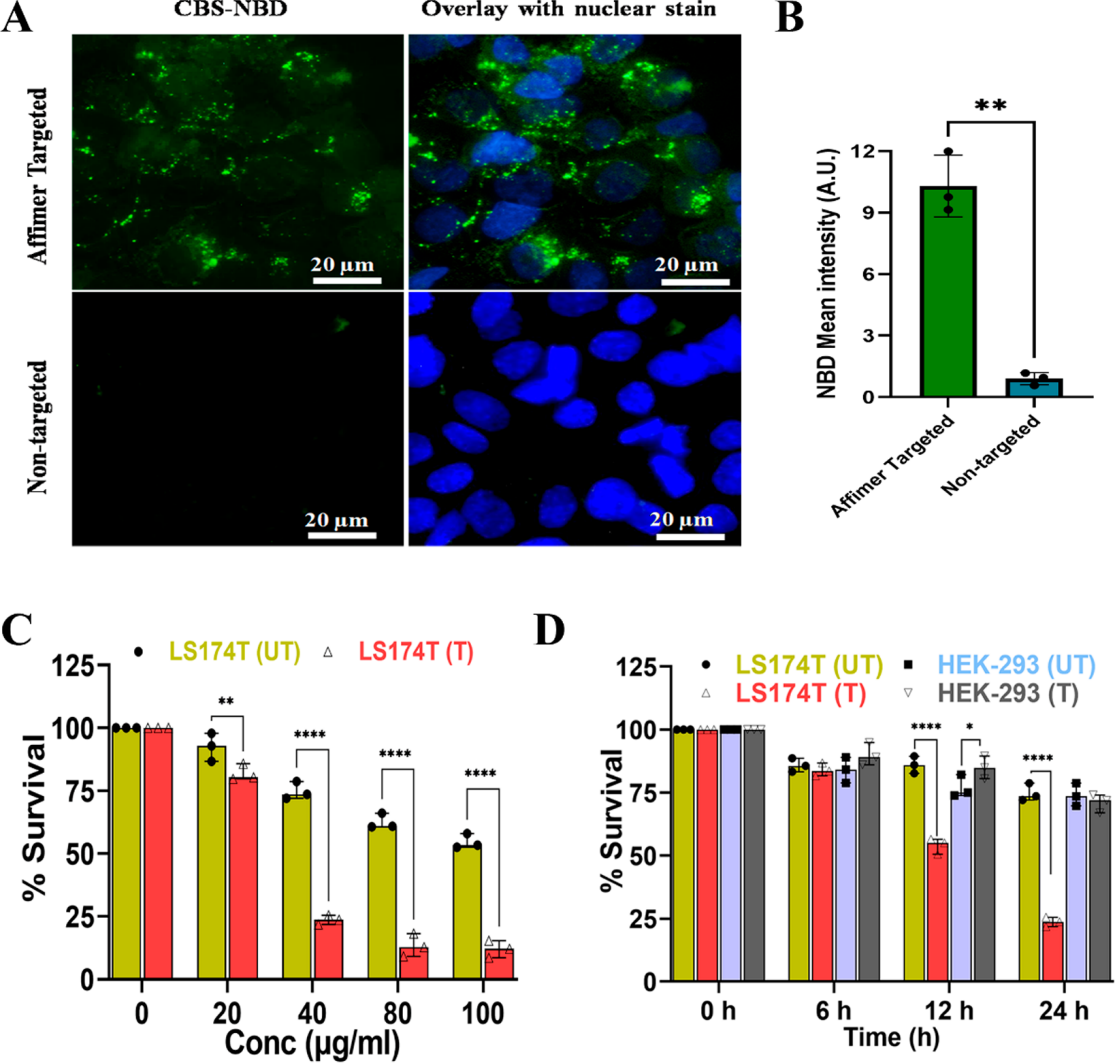
*In vitro* localization and targeting efficiency of Affimer labeled cubosomes. (A) Localization of fluorescently labeled cubosomes (Cbs-NBD) in LS174T with and without Affimer tagging. Cbs-NBD was observed at the cytoplasmic region of the cells post-24 h treatment when tagged with Affimer, whereas CBS-NBD uptake was negligible in the absence of Affimer, showing specificity of Affimer tagged cubosomes toward CEA expressing LS174T cells. (B) Quantitative analysis of Cbs-NBD (i.e., green florescence) shows a significant increase of 9.5 fold (**p < 0.01 using Welch’s nonparametric *t* test) when Affimers are tagged to the cubosome compared to nontargeted cubosome in LS174T cells. (C) Cytotoxicity evaluation of Cbs-Cu, i.e., untargeted represented as “UT” and Cbs-Cu-Af i.e Affimer targeted represented as “T” in LS174T cells at concentrations between 0 and 100 μg/mL. There was a significant decrease (****p < 0.0001, **p < 0.01 using two-way ANNOVA) of survivability of the cells when CuAc was delivered via Cbs-Cu-Af. This observation was noted with dose starting from 40 μg/mL. (D) Cell viability of LS174T and HEK-293 cell lines with 40 μg/mL of Cbs-Cu “UT” and Cbs-Cu-Af “T” at various time points up to 24 h. Significant reduction (****p < 0.0001 using three-way ANNOVA) of survivability in LS174T cells is evident when treated with Cbs-Cu-Af but has a negligible effect on HEK-293 cells as they lack the CEA expression. Without Affimer “UT” there was negligible toxicity in either of the cell lines.

Affimer tagged cubosomes labeled with the green fluorescent lipid NBD-PE (Cbs-NBD) were added to CEA expressing LS174T cells and were found to be endocytosed, as suggested by the green fluorescence observed around the cell nucleus, in the cytoplasmic region of the LS174T cells after a period of 24 h (Figure 3A,B). On the contrary, fluorescent cubosomes that where not Affimer tagged showed little uptake by the LS174T cells during the time frame of the experiment. It has been shown that PEGylation of nanoparticles can hinder cell–nanoparticle interactions due to steric hindrance, and consequently a target moiety is needed to overcome this barrier and promote uptake via receptor mediated endocytosis.^40,41,53^ Our results suggest that Affimer tagged cubosomes show promise in selectively delivering cargo to CEA expressing cells.

### Affimer Tagged Cubosomes Show Selective Toxicity to Colorectal Cancer Cells: *In Vitro* Studies

Monolayer 2D cultures of LS174T and HEK-293 cells were chosen for studying the drug targeting efficiency of Affimer tagged cubosomes. Bare cubosomes were studied for their biocompatibility in cells which concluded no cytotoxicity in both cell lines at a concentration of up to 100 μg/mL (Figure S11). To identify an optimum concentration of CuAc (5 wt %) loaded cubosomes, LS174T cancer cells were initially screened under varying concentrations (0–100 μg/mL) of Cbs-Cu (with and without Affimer) for a period of 24 h. A concentration of 40 μg/mL showed a significant decrease in cell viability, with the Affimer targeted and nontargeted cubosomes showing a survival rate of 21 ± 6% and 75 ± 4%, respectively (Figure 3C). Further cytotoxicity studies were performed at 40 μg/mL in both the cell lines (with and without Affimer tagging) at varying time points over a period of 24 h. The noncancerous HEK-293 cells showed no significant reduction in cell viability (80 ± 5%) when treated with both targeted and nontargeted Cbs-Cu (Figure 3D). Contrastingly, although LS174T cells showed a high cell viability when treated with nontargeted cubosomes, Affimer tagged cubosomes showed a significant drop in cell viability (52 ± 4%) after 12 h (Figure 3D). This result is in agreement with our cubosome localization study above and suggests that Affimer tagged, drug loaded cubosomes are taken up by cells within a 6–12 h period, whereas noncancerous CEA negative cells displayed minimal uptake and cytotoxicity and show promise in targeted delivery to CEA expressing cells with low toxicity to normal cells. This is the first demonstration of Affimer-directed specific cancer cell death using drug-loaded cubosomes. Cell death of the LS174 CEA-expressing cell line treated with targeted cubosomes was shown to be mediated by apoptosis (Figure S12). A clear difference is seen in Affimer tagged and untagged cubosomes in efficiency of targeting, which proves Affimers are active even after tagging on cubosomes.

**Figure 4:**
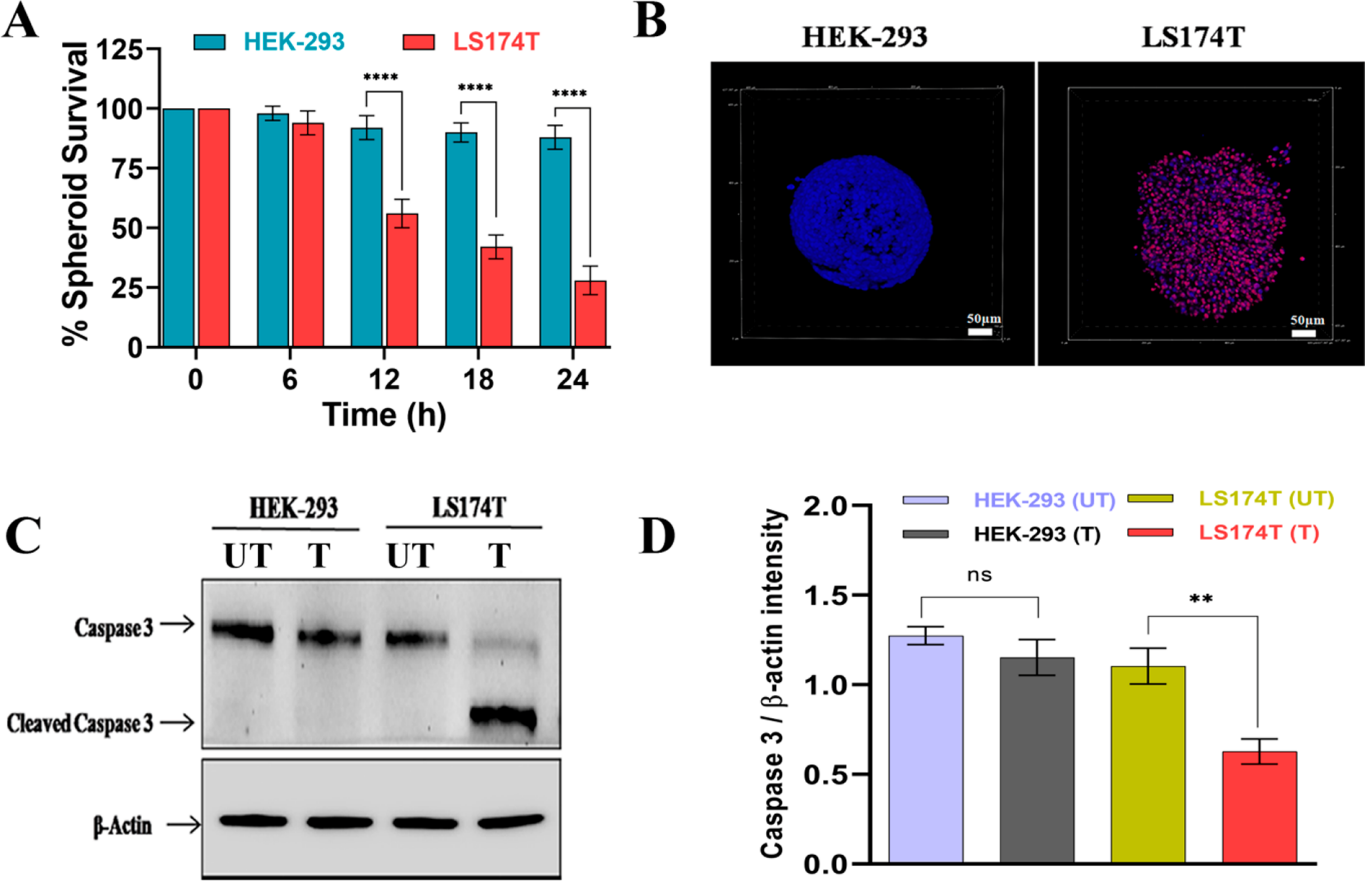
3D spheroid study of Affimer targeting. (A) Survivability study of 40 μg/mL Cbs-Cu-Af on 3D spheroids of LS174T and HEK-293 up to 24 h of treatment. The survivability was measured by using the intensity of red (propidium iodide) and blue (Hoechst 33342) fluorescence denoting dead and live cells, respectively. LS174T spheroids had a significant reduction (****p < 0.0001 using two-way ANNOVA) in survivability after 12 h whereas negligible effects were observed in HEK-293 spheroids even after 24 h. (B) Confocal images of the spheroids after 24 h treatment with 40 μg/mL Cbs-Cu-Af showing the above observation. (C) Whole cell lysate from the spheroids after 24 h treatment of Cbs-Cu with Affimer tagged delivery “T” or without Affimer “UT” was analyzed for apoptosis using Western blot of Caspase 3 marker. (D) Intensity plot for caspase 3 from the blot. From the band intensity measurement it was evident that in the case of LS174T spheroids there was a significant decrease (**p < 0.01 using unpaired *t* test) in full length caspase indicating apoptosis only in the LS174T cells upon Affimer tagged delivery of Cbs-Cu whereas no significant sign of apoptosis in the case of HEK-293 spheroids.

The 3D tumor spheroids are considered to be much more relevant models to evaluate drug efficacy and mimic solid tumors *in vivo* as compared to conventional monolayer 2D cultures.^54,55^ The cytotoxicity of Cbs-Cu-Af (40 μg/mL) on spheroid models of both HEK-293 and LS174T cell lines was studied, and it was observed that after 24 h of treatment HEK-293 spheroids showed a survivability of 88 ± 5% whereas a significant drop of 30 ± 6% was noted in the case of LS174T spheroids (Figure 4A,B). It has been shown that 3D spheroids can be more resistant to drugs and delivery vehicles compared to 2D cultures.^9,56^ Although the survival rate of LS174T spheroids is slightly higher than the 2D culture data, Cbs-Cu-Af cubosomes are effective in specific targeting in the spheroid models. This finding was further validated with Western blot studies using a caspase 3 marker and confirmed that Cbs-Cu-Af cubosomes induced apoptosis^38^ upon targeted delivery in LS174T cells (Figure 4C,D).

### Affimer Functionalized Cubosomes Show Targeted Release of Payload in Tumors *In Vivo*

**Figure 5:**
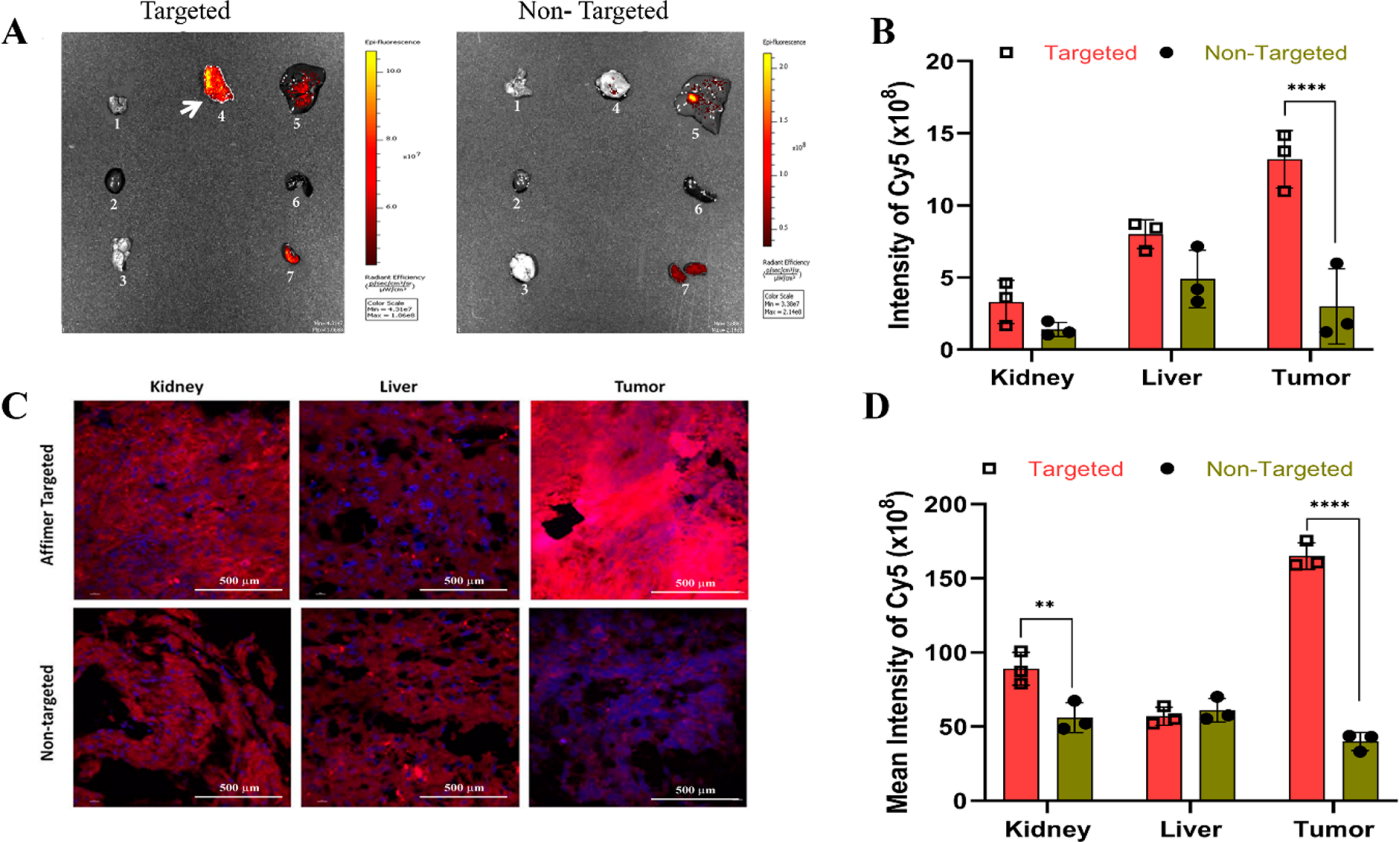
*In vivo* tracking of cubosomes by Cy5 fluorescence. (A) IVIS images of whole organs, namely lung, heart, brain, tumor, liver, spleen, and kidney as numbered from 1 to 7 in the image, showing uptake of Cy5 in various organs in the Affimer targeted and nontargeted groups of mice after 72 h of administration. As evident, Cy5 accumulation is maximum in the tumor for the targeted group compared to the nontargeted group. Note the use of different scales in the two images that maximizes the dynamic range of detection; the scale used for the targeted image is less sensitive and therefore underrepresents the relative intensities when compared to the nontargeted. (B) Quantitative fluorescent intensities of the IVIS image in the kidney, liver, and tumor of both groups. Significant increase (****p < 0.0001 using two-way ANNOVA) of Cy5 was observed in tumor of group administered with Affimer targeted delivery whereas nontargeted group showed the maximum accumulation in the liver. (C) Tissue uptake of Cy5 was studied in 5 μm tissue sections of kidney, liver, and tumor of both groups using confocal microscopy (D) along with their quantitative mean fluorescence intensities. Tumor tissue uptake of Cy5 was found to be maximum (****p < 0.0001 using two-way ANNOVA) in the Affimer targeted (Cbs-Cy5-Af) group, whereas in the nontargeted group (Cbs-Cy5) maximum uptake was shown in the kidney and liver.

Having shown that Cbs-Cu-Af cubosomes could preferentially target CEA positive LS174T cells to deliver the drug, their ability to target tumors in *in vivo* models was investigated. We used subcutaneous (heterotopic) xenograft tumors of LS174T cells as our model, since subcutaneous models provide a suitable environment for testing pharmacology and activity of novel agents.^57^ Fluorescent dyes have been either been encapsulated or tagged on cubosomes to study their localization *in vivo*.^9,25^ Here we chose a far-red-fluorescent hydrophobic Cy5 dye to study the localization of Affimer tagged cubosomes in *in vivo* models. Whole organs of mice (brain, liver, kidney, spleen, heart, and lung along with the tumor) were quantified for their Cy5 fluorescence by *ex vivo* IVIS imaging upon delivery of the Cy5 loaded Affimer tagged cubosomes (Cbs-Cy5-Af), with a suitable control (Cbs-Cy5) at various time points (Figure S13A,B). As observed in Figure 5A, after a period of 72 h post-administration, the fluorescence intensity indicated the accumulation of Cy5 mainly in the tumor regions (indicated by an arrow) of the Affimer targeted mice group (Cbs-Cy5-Af). For the control group (Cbs-Cy5 cubosomes), the concentration of dye was noted to be maximum in the liver. As observed in our *in vitro* study (Figure 3), we hypothesize that the Affimer tagged cubosomes are preferentially taken up by the tumor cells via receptor mediated endocytosis, followed by an interaction with the endolysosomal compartment leading to the release of the payload.^58^ A significant increase in Cy5 intensity was noted in tumor tissues of the targeted group as compared to nontargeted group (Figure 5B). A high level of accumulation of therapeutic nanoparticles in the liver has been noted as a common bottleneck to their applications.^59^ A few studies have shown that cubosomes can improve the efficacy of drugs loaded in them; however, there is a scarcity of knowledge on how these nanoparticles behave *in vivo* as well as their biodistribution. The handful of studies that have reported on this have shown that biodistribution depends on the route of administration, with lipid nanoparticles administered intravenously preferentially accumulating in the liver, spleen, and kidneys.^3,11,16^ These results differ to our findings, and we attribute the preferential accumulation in the tumor to active targeting using Affimer tagged cubosomes. Moreover, although it is known that smaller nanoparticles are absorbed by the kidneys, heart, lung, and brain in addition to the liver and spleen,^60^ our fluorescent cubosomes showed essentially no accumulation in these organs, indicating an absence of nonspecific cubosome absorption in either of the mice groups (targeted and nontargeted) as seen in the IVIS images (Figure 5A,B). To further validate these findings, tissue sections of kidney, liver, and tumor were examined for Cy5 uptake by confocal microscopy (Figure 5C). Similar to the above observations, Cy5 absorption was found to be 5–7-fold higher in the tumor tissue of the targeted group (Cbs-Cy5-Af) as compared to the nontargeted group administered with Cbs-Cy5 (Figure 5D). A 3-dimensional reconstruction of the tumor tissue sections of the targeted and nontargeted groups is shown in Figure S13C. Similar to our *in vitro* targeting results, Affimer tagged cubosomes could selectively deliver the payload to the tumor tissue and show promise as novel nanocarriers with proven biosafety and biodistribution features.

### Cubosomes Have Promising Therapeutic Efficacy upon Targeted Delivery to the Tumor Xenograft

**Figure 6:**
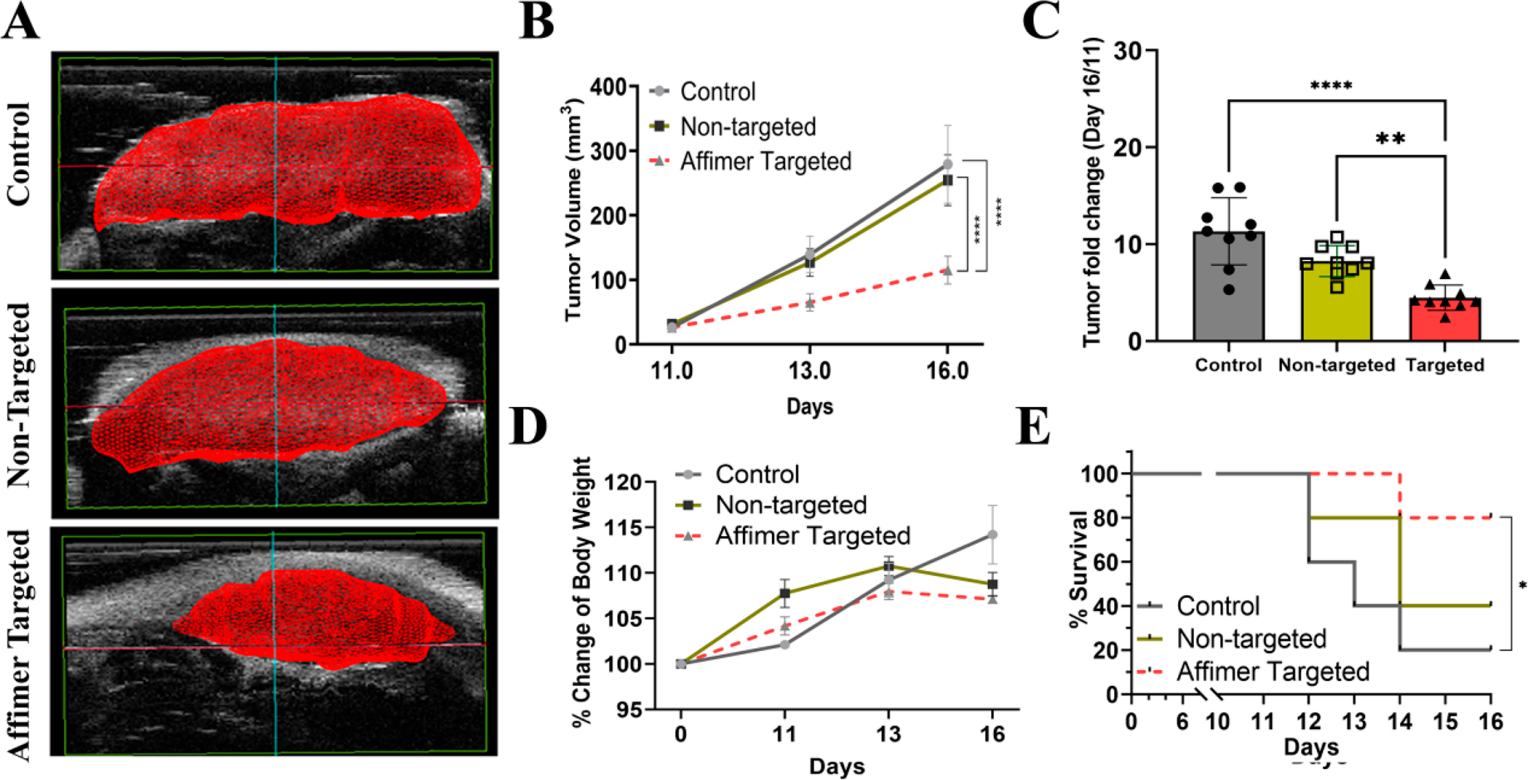
Efficacy of Affimer mediated drug delivery *in vivo*. (A) 3D reconstruction of ultrasonography (USG) measured tumor volume of the three groups, i.e., control (saline), nontargeted (Cbs-Cu), and Affimer targeted (Cbs-Cu-Af) administration showing tumor growth restriction in the case of the Cbs-Cu-Af. (B) Quantitative data of tumor volume as recorded in the three groups by USG on day 11, 13, and 16 where it is evident that after the first dose of administration on day 11, tumor growth was restricted in the targeted group. Here, by use of the unpaired *t* test, statistical analysis of day 16 shows a significant difference (****p < 0.0001) between targeted and control group as well as (****p < 0.0001) the targeted and nontargeted group. (C) Data representing the fold change in tumor volume from day 11 to day 16 in the groups which shows post-targeted delivery of Cbs-Cu resulted in significant reduction (using one-way ANNOVA) of tumor growth compared to control (****p < 0.0001) as well as nontargeted group (**p < 0.01). (D) Change in the body weight of mice in the three groups during the study were measured and no reduction of body weight (as a sign of toxicity) was noted. (E) Survival rate of mice in the groups represented by a Kaplan–Meier curve as per the tumor volume reaching the permissible limit and hence euthanized. Using the logrank test, a significant increase (*p < 0.05) of survivability was noted in targeted group compared to the control group. The survivability of Affimer targeted group was 80% on day 16, whereas in control and nontargeted they were 20% and 40%, respectively.

The key indicators for successful targeted delivery of a chemotherapeutic drug in mice include restricted tumor growth, increase survivability, and low signs of organ toxicity.^40,41^ Ultrasound imaging was used to record the tumor volumes of mice administered with saline (control), Cbs-Cu (nontargeted), and Affimer targeted (Cbs-Cu-Af) cubosomes and were recorded as a function of time (Figure S14). The 3-dimensional reconstruction of the tumor volume on day 16 showed significant inhibition of tumor growth in the Affimer targeted group (Figure 6A). The mean tumor volume was noted to be 115 ± 52.0, 254 ± 96.0, and 279 ± 147.0 mm^3^ for the Affimer targeted, nontargeted, and control groups, respectively (Figure 6B). The fold increase in tumor volume was calculated with reference to the treatment start V0 (day 11). The mean fold increase in tumor volume was found to be 4.4 ± 2.5, 8.2 ± 2.1, and 11.3 ± 7.3 for Affimer targeted, nontargeted, and control groups, respectively (Figure 6C). As evident from Figure 6C, data scatter in the case of the Affimer targeted group was found to be the least, which is in accordance with reports on *in vivo* studies of cisplatin targeted delivery by using hyaluronic acid,^40^ or a paclitaxel formulation targeted by a RGD peptide,^41^ where the tumor volume was recorded and a size reduction of about 66% was observed in the targeted group. One of the routine assessments to evaluate the toxicity arising from drug administration is indicated by a change in body weight or a loss of weight in mice.^40,61^ The body weight of mice was recorded at regular intervals during the study. Results showed a gradual increase (although within 10%) in the control group with minimal effects in the other two groups, thereby indicating a minimal effect on the welfare of the mice upon treatment (Figure 6D). As shown in Figure 6E, the survivability of CRC tumor xenograft bearing mice was assessed in the three groups post-treatment; survival was denoted as the tumor volume reaching the maximum permissible diameter of 17 mm. Survivability and the extent of drug toxicity could be directly correlated to the efficacy of the therapeutic.^41^ A successful targeted delivery would result in enhanced welfare of the animal survival ensuring a minimal sign of toxicity.^41,61^ In our study, the survival of the control group was reduced to 40% on day 13 and 20% on day 16. The group of mice administered with Cbs-Cu-Af showed a maximum survival of 80% on day 16 as the tumor growth was restricted after targeted delivery of CuAc, whereas the group injected with nontargeted cubosomes (Cbs-Cu) showed a 40% survival. An improved survivability in the nontargeted group could be attributed to the enhanced permeability retention effect (EPR) due to the tumor vasculature.^62^ Thus, these data indicate that the targeted delivery of Cu-Cb-Af has a positive impact on the mice heath and restricted the tumor growth. It would also be of value to assess in the future this effect in orthotopic models, especially of locally disseminated disease,^63^ for which systemic targeted therapeutics have real potential to impact on clinical outcomes.

**Figure 7:**
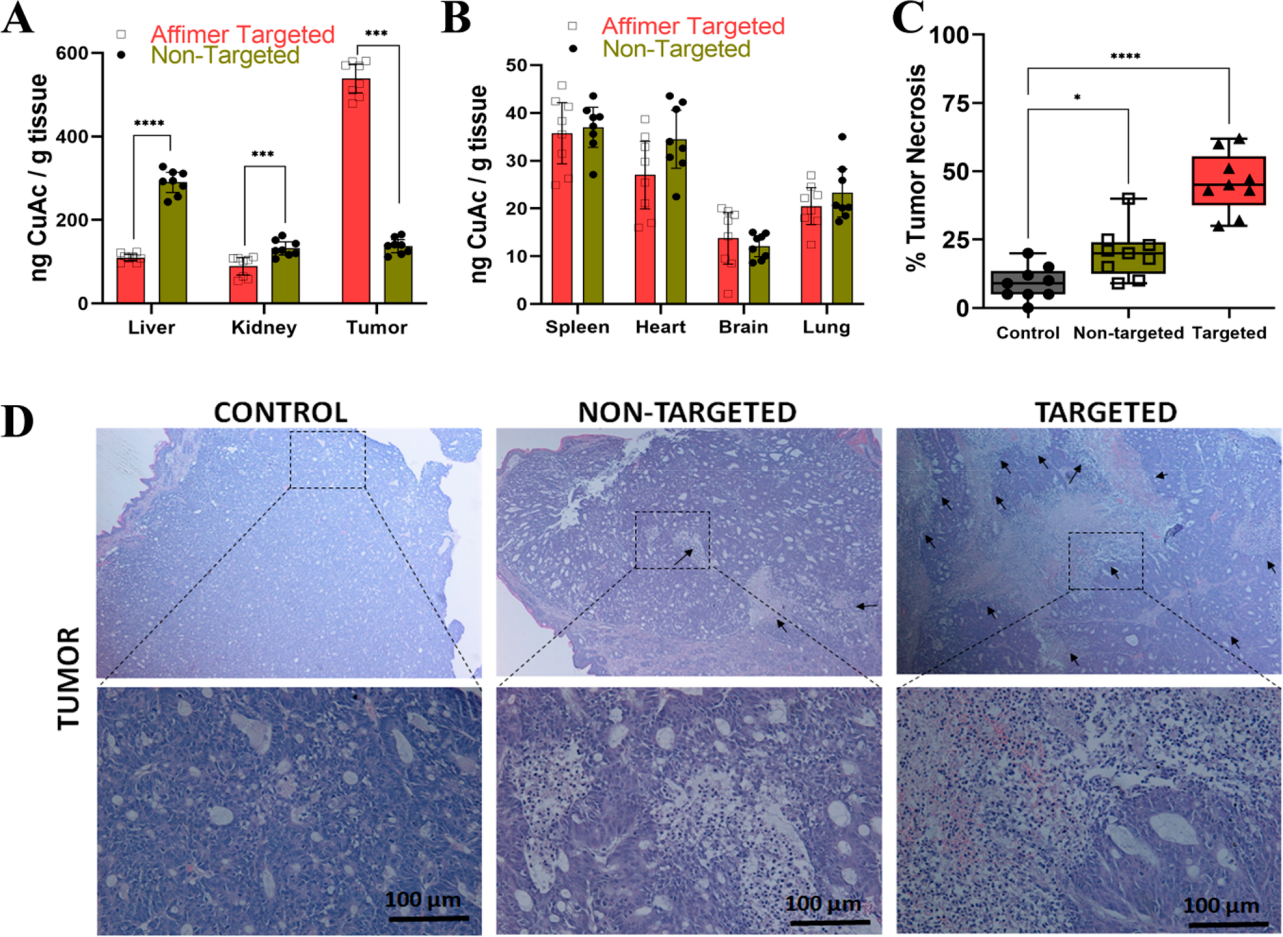
Biodistribution of CuAc in organs and biosafety study upon Affimer targeted delivery of CuAc encapsulated cubosomes. ICP-OES data of CuAc uptake in the (A) liver, kidney, and tumor of the nontargeted (Cbs-Cu) and Affimer targeted (Cbs-Cu-Af) groups. A significant increase (****p < 0.0001 using two-way ANNOVA with Bonferroni’s correction) in CuAc uptake in tumor tissue of the Affimer targeted group was noted whereas accumulation was highest in the liver for nontargeted.(B) Tissue absorption of CuAc measured from ICP-OES in other organs such as spleen, heart, brain, and lung was negligible and well below the limits of any safety concern. (C) Quantitative tumor tissue necrosis data as studied by haematoxylin and eosin (H&E) staining along with (D) the microscopy images of tumor tissue sections of the three groups. A significant increase (****p < 0.0001) in tissue cell death was noted for the Affimer targeted group compared to the control which indicates CuAc having maximum effect on tumor upon targeted delivery.

Drug safety is assessed based on its effect on vital organs,^40,41,64^ and one such method to analyze the toxicity is an in-depth tissue study. Inductively coupled plasma optical emission spectrometry (ICP-OES) has been used in the past to determine the accumulation of metal based drug such as platinum in various organ.^60,65^ Here we used ICP-OES to estimate Cu uptake as a reference to CuAc distribution in homogenized organ samples, including the liver, kidney, spleen, heart, brain, and lung along with tumor. In the targeted delivery of the Cbs-Cu-Af mice group, the maximum uptake of CuAc was noted in the tumor tissue, whereas in the nontargeted group, major uptake was found to be in liver followed by moderate amounts in the tumor and kidney. A comparison between targeted and nontargeted groups suggests an approximately 4.5-fold increase in CuAc uptake in the tumor tissue of the targeted group, which is similar to our findings on the Cy5 distribution as shown in Figure 5. The uptake of CuAc was approximately 5-fold higher in the tumor compared to the kidneys and liver for the targeted groups of mice (Figure 7A). Thus, these data indicate specificity of Cu-Cb-Af cubosomes toward tumor cells with up to 550 ng of CuAc per gram of tissue accumulation compared to nontargeted Cu-Cb cubosomes, which had about 120 ng of CuAc per gram of tissue. Other tissues of spleen, heart, brain, and lung had negligible CuAc accumulation (Figure 7B).

Haematoxylin and eosin staining of tissue sections is common practice to study toxicity associated with drugs.^40,66^ Tumor cell death was studied in the tissue sections for all three groups (Figure 7C,D).The percentage of dead cells in the tumor tissue were measured in all groups and showed that targeted delivery resulted in a significant increase in tumor cell death (Figure 7C) compared to nontargeted delivery which was almost 2.5–3 times higher. Yet again, the significant cell death in the nontarget tumor group could be due to the EPR effect. Similar to the study for doxorubicin delivery in colon cancer by Wei et al.,^66^ we further confirmed the safety of this drug administration by examining tissue sections of the heart, liver, and kidney of the three groups by histology. As seen in Figure S15, no significant signs of cell death (i.e., organ toxicity) was observed in both targeted and nontargeted delivery groups which could be attributed to the absence of CEA expression in those cells. In addition, tissue sections of lung, brain, and spleen (Figure S16) of targeted and nontargeted groups were also examined, and no tissue cell death was noted. Hence, the data and observations could be correlated to the biosafety of the drug loaded nanocarrier.

## Conclusion

We have developed monoolein-based cubosomes, with an internal nanostructure based on space group *Im*3*m*, that have been functionalized for the first time with an Affimer protein via copper-free click chemistry to actively target carcinoembryonic antigen expressing colorectal cancer cells. The cubosomes could selectively target the cancer cells both *in vitro* (2D monolayer cultures and 3D spheroid models) and *in vivo*. Targeted cubosomes, loaded with the model anticancer drug copper acetylacetonate, showed high efficacy in the tumor tissue of mouse xenografts and resulted in significantly restricting tumor growth, a high survival rate compared to the control groups, no signs of toxicity, and low nonspecific tissue absorption in other vital organs. Because of the limited studies on actively targeted, drug loaded cubosomes, as well as their performance and efficacy *in vivo*, we hope these results will add to the growing body of knowledge of cubosomes as promising delivery vehicles for cancer therapy and shed light into their biodistribution and efficacy *in vivo* that may aid to clinical translation of these promising lipid nanoparticles.

## Materials and Methods

### Clickable Cubosome Preparation and Payload Encapsulation

Cithrol GMO (MO) was a kind gift from Croda (Croda Personal Care, Goole, UK) and is a commercial version of monoolein, containing a minimum of 92% monoester and a maximum 8% diester. DSPE-PEG-2000 azide (DPA) was purchased from Avanti Polar Lipids (AL, USA) and Pluronic F-127 from Sigma-Aldrich (Gillingham, UK). Bare cubosomes were prepared by using various ratios of MO (90–95% w/w):DPA (5–10% w/w) codissolved in chloroform (Merck, Branchburg, NJ) and dried under nitrogen gas. To ensure all solvent had evaporated, the dry lipid films were left in a desiccator overnight at room temperature. The lipid films were hydrated with phosphate-buffered saline (PBS; Sigma-Aldrich Gillingham, UK) containing Pluronic F-127. The concentration of F127 was varied between 2 and 7 wt % of the MO. Nanoparticle dispersions were prepared by tip sonicating the sample in 1 mL of buffer by using a Q125 sonicator (Qsonica, USA) for 30 min in pulse mode (1 s pulse on, 1 s off) at 80% amplitude in an ice bath. The resultant cubosomes were then passed through a mini extruder (Avanti Polar Lipids, USA) using a 100 nm pore size polycarbonate membrane (Whatman, USA). For drug loaded cubosomes, copper acetylacetonate (CuAc; Merck, USA) was dissolved in chloroform and added in various amounts (1–5% w/w) to the codissolved lipid mixtures. The same steps described above for bare cubosomes were then followed. The drug loaded cubosomes were placed in Slide-A-Lyzer cassettes (2K MWCO, Thermo Scientific, UK) in PBS at 25 °C to remove any free CuAc. The external PBS was changed at regular interval while performing the dialysis.

For studying cubosome localization by fluorescence *in vitro*, 0.5% w/w of 1,2-dioleoyl-*sn*-glycero-3-phosphoethanolamine-*N*-(7-nitro-2-1,3-benzoxadiazol-4-yl) (ammonium salt) (18:1-NBD PE; Avanti Polar Lipids, USA) was codissolved with the lipid mixtures in chloroform before the drying step. For the *in vivo* fluorescent studies, 2% w/w of Cy5 dye (MedChemExpress, USA) was codissolved with the lipid mixtures in chloroform. As with the CuAc loaded cubosomes, Cy5 labeled cubosomes were loaded in dialysis cassettes to remove any free dye.

Inductively coupled plasma optical emission spectrometry(iCAP 7600 ICP-OES Analyzer, Thermo Scientific, UK) equipped with a 240-position Cetac autosampler was used to estimate the amount of copper as an indicator of CuAc encapsulated in the cubosomes by using a known concentration of copper solution as a standard curve. The encapsulation efficiency (%) was calculated via eq 1:
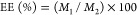
1where *M*_1_ represents the weight of drug encapsulated in mg (obtained from ICP-OES) and *M*_2_ represents total drug added (mg) to the cubosomes.

### Affimer Cloning and Production

Anti-CEA specific Affimer clones were identified by using a “phage display library” method as recently published by Shamsuddin et al.^35^ Out of the three CEA binding Affimers identified, clone II and III were chosen for this study having 9 and 10 distinct amino acid residues at the variable region, respectively, in clone II and clone III. Based on a 50 mL of working volume, the yield of the Affimers were noted to be 8.3 and 6.27 mg for clone II and III, respectively,^35,36^ with corresponding molecular weights noted to be 12.5 and 12.6 kDa (Figure S4A). The associated DNA from the positive clones were sequenced. The coding region of the selected Affimers was amplified by PCR during which a cysteine codon at the C-terminal was inserted for the ease of functionalization. The Affimer coding sequence was inserted into the pET11a vector by using two restriction enzymes NheI-HF and NotI-HF, and then Affimer production was done in BL21 (DE3) *E. coli* cells as previously reported.^32^ The E. coli cells were grown in Luria–Bertani broth medium containing 100 μg/mL of carbinicillin until the growth absorbance value was 0.8 at 600 nm. Then cells were induced with 0.1 mM IPTG and incubated at 25 °C for 6 h. The cells were harvested by centrifugation and lysed, and the His_8_ tagged Affimers were purified on Ni2+-NTA affinity chromatography (Merck, Branchburg, NJ). The binding efficiency of these Affimers to the CEA receptor was thoroughly studied and confirmed by using surface plasmon resonance as reported by Shamsuddin et al.^35^ The surface plasmon resonance showed that the Affimers demonstrated a high binding affinity toward CEA (*K*_D_ value for clone II: 15.3 ± 0.37 nM; *K*_D_ value for clone III: 34.4 ± 16 nM) (Figure S4B).

### Functionalization of Cubosomes with Affimers

Affimers were attached to the cubosomes by using DBCO-maleimide (Kerafast, Inc., Boston, MA) click coupling chemistry. Briefly, 2 mL (0.5 mg/mL) of Affimer clone II and clone III were reduced by using 150 μL (5.7 mg/mL) of TCEP-HCl (Merck, Branchburg, NJ) for 90 min to remove any dithiol-linked dimers. The reduced Affimers were incubated with 4 mM DBCO-maleimide for 2 h to attach DBCO, and then 100 mg/mL azide containing cubosomes was added to allow click coupling to occur and incubated for overnight at room temperature. The final product was dialyzed for 24 h in 1x PBS by using Slide-A-Lyzer Dialysis Cassettes, 5K MWCO (Thermo Scientific, Waltham, MA) to remove unreacted Affimers. FTIR spectroscopy (Platinum ATR, Model Alpha, Bruker, UK) was used to confirm the covalent conjugation between the azide group of cubosomes and the DBCO group attached to the Affimers.

### Small-Angle X-ray Scattering (SAXS)

The internal nanostructure of the cubosomes was probed with SAXS. The measurements were done at 25 and 37 °C (5 min equilibration at the desired temperature with and accuracy of ±0.1 °C) for all three samples: bare cubosomes, drug loaded cubosome (Cbs-Cu), and Affimer tagged drug loaded cubosomes (Cbs-Cu-Af). Synchrotron SAXS measurements were performed on beamline I22 at Diamond Light source. The synchrotron beam was tuned to a wavelength of 0.69 Å with a sample-to-detector distance of 3.7 m, and the 2-D SAXS patterns were recorded on a Pilatus 2M detector. SAXS experiments were also conducted on a lab-based Xeuss 3.0 (Xenocs, France) beamline equipped with a liquid gallium MetalJet X-ray source (Excillum, Sweden) which has an energy of 9.2 keV, corresponding to a wavelength of 1.34 Å. 2-D SAXS patterns were recorded on a Eiger2 R 1M detector (Dectris, Switzerland), and the sample-to-detector distance was set to 0.8 m, giving a *q* range of 0.01–0.4 Å^–1^. Silver behenate (*a* = 58.38 Å) was used to calibrate the SAXS data. SAXS images were analyzed by using the IDL-based AXcess software package or the DAWN software.^67,68^

### Dynamic Light Scattering (DLS)

The hydrodynamic diameters, i.e., the particle sizes of all cubosome samples, were measured at 25 °C by using a DLS instrument (Zetasizer Nano ZS90, Malvern Panalytical, Malvern, UK) at a fixed backscattering angle of 173°. The refractive index of the cubosomes was set to 1.46 (pure MO) with an absorbance of 0.10. The refractive index of the dispersant (PBS) was set to 1.332 with a viscosity of 0.9053 cP.

The sizes of Cbs, Cbs-Cu, and Cbs-Cu-Af samples were measured by adding 100 μL of cubosomes into 900 μL of PBS in a 3 mL cuvette. The instrument equilibration time was set for 120 s at 25 °C, and samples ran for 10 cycles with 10 measurement in each cycle. For zeta-potential measurements,100 μL of Cbs-Cu-Af was added to 900 μL of water (with a resistivity of 18.2 MΩ·cm at 25 °C) in a disposable zeta cuvette, and the sample was equilibrated for 120 s at 25 °C. The instrument was set to run 20 cycles with 10 measurements in each cycle.

### Transmission Electron Microscopy (TEM)

Morphological analysis of the Cbs-Cu-Af cubosome was done by using a high-resolution transmission electron microscope (FEI Tecnai TF20) fitted with field emission gun TEM/STEM along with a HAADF detector. For this study, a 200 mesh carbon film coated on a nickel grid (EM Resolutions, UK) was used. Ten microliters of Cbs-Cu-Af (10 mg/mL) in PBS was added on the grid, and any excess droplets were soaked up by using an absorbent filter paper. The grid was left in a desiccator to dry for 24 h. The sample was imaged at 13000× magnification at an accelerating voltage of 300 kV. The image was captured by using a Gatan Orius SC600A CCD camera. Further images were analyzed by using Fiji ImageJ software (NIH, USA). The same sample was analyzed by energy dispersive X-rays equipped in the FEI Tecnai TF20 (Oxford Instruments INCA 350 EDX system/80 mm X-Max SDD detector) to study the presence of CuAc in the cubosome (copper as a marker). The advantage of using a nickel grid over a standard copper grid in this study was to eliminate any background noise of copper during this EDX study.

### Cryogenic Transmission Electron Microscopy (Cryo-TEM)

Cubosomes at a concentration of 79 mg/mL were used for the morphological characterization using cryo-TEM. Three microliters of sample was deposited to freshly glow discharged Cu QUANTIFOIL grids (R2/R2, 300 mesh) with a holding time of 30 s. The carbon coated grids were glow discharged at 10 mA for 20 s and blotted for 6 s (blotting force of 7 at 25 °C under 100% relatively humidity). The grids were subsequently plunged into liquid ethane by using a Vitrobot mark IV (Thermo/FEI). A Titan KRIOS microscope (Thermo Fisher Scientific, USA) with an accelerating voltage of 300 kV and a defocus value of −4 μm was used to image the cubosomes at a magnification of 47000 which has a pixel size of 1.76 Å. Image processing and analysis were done by using Fiji. Indexing of the cubosome was determined by obtaining the *d*-spacing of each reflection in the FTT by using TrackMate.^69^

### Cell Culture

CRC cell line LS174T and noncancerous HEK-293 cells were originally obtained from ATCC and were subjected to mycoplasma testing and STR typing (Source Bioscience, UK) before use. Cells were grown in DMEM (Thermo Scientific, Waltham, MA) growth medium supplemented with 10% (v/v) fetal calf serum (FCS; Thermo Scientific, Waltham, MA) and penicillin/streptomycin (Thermo Scientific, Waltham, MA) at 100 units/mL. All cells were cultured in a humidified incubator with 5% CO_2_ at 37 °C. Cells were maintained, and experiments were conducted at cell densities that allowed exponential growth or otherwise mentioned.

### Immunofluorescence Assay for Detecting of CEA Expression

LS174T and HEK-293 cells were grown in complete growth medium for 48 h, then washed in PBS, and fixed with 4% (w/v) paraformaldehyde (Merck, Branchburg, NJ) in PBS at room temperature for 10 min. The fixed cells were further washed with PBS and permeablized with 0.2% (v/v) Triton X-100 (Merck, Branchburg, NJ) in PBS on an ice bath for 10 min. Cells were then washed with PBS several times and blocked with 5% (v/v) FCS in PBS for 1 h in an ice bath. Subsequently, the cells were incubated with mouse anti-human IgG CEA monoclonal antibody (cat. no. MA5-14675, Thermo Scientific, USA) at 1 μg/mL overnight at 4 °C. The following day, several washes were performed with wash buffer, comprising 0.5% (v/v) FCS and 0.05% (v/v) Tween-20 in PBS. Cells were then incubated with Alexa Fluor 488 labeled secondary antibody (cat. no. A-11001, Thermo Scientific, USA) at 1 μg/mL for 1 h at room temperature in the dark. Cells were then washed with wash buffer several times and mounted with Fluoromount-G mouting media with DAPI (Thermo Scientific, USA) before analyzing them under a confocal microscope (Nikon A1R LSM), with a 405 nm laser for DAPI with excitation and emission wavelengths of 407 and 450 nm. For CEA expressing detection, a 488 nm laser was used with excitation and emission wavelengths of 488 and 525 nm, respectively. Images were captured by using a 100× objective with a numerical aperture of 1.4. The images were analyzed by using the NIS-element viewer software (ver. 5.20.01).

### Western Blot Analysis for CEA Protein Expression and Apoptotic Markers

Western blots were performed as detailed in our previous work.^38^ Briefly, gel electrophoresis was performed for 90 min at 120 V on a 4–12% precast polyacrylamide gel (Bio Rad, Hercules, CA). The proteins were then transferred to a PVDF membrane and blocked with 5% (w/v) nonfat skimmed milk in TBST (Tris buffered saline with 0.1% Tween-20) for 1 h. The membrane postblocking was labeled with respective primary and secondary antibodies and further imaged under a chemi-doc instrument (Biorad, USA) after incubating with Pierce ECL reagent.

### Confocal Microscopy

Confocal microscopy was use to localize cubosomes *in vitro*. LS174T cells were seeded in glass coated chambered slides (Thermo Scientific, USA) overnight for 18 h. Next, cells were treated with 20 μg/mL of NBD-PE cubosomes with and without Affimer tagging for 24 h. Then cells were gently washed with PBS and incubated with 5 μg/mL Hoechst 33342 for 15 min before imaging the cells under a confocal microscope with 100× objective lens and numerical aperture of 1.4. For nuclear staining (Hoechst 33342) a 405 nm laser was used at excitation and emission wavelength of 407 and 450 nm. For cubosome detection a laser of 488 nm was used with excitation and emission of 488 and 525 nm, respectively. Images were captured by using the Galvano scanning mode and analyzed with the NIS-element software (ver. 5.20.01).

### *In Vitro* Targeting Studied in Monolayer Culture and 3D Spheroids

LS174T and HEK-293 cells were seeded in 24 well culture plates in complete DMEM growth media at densities of 2.5 × 10^4^ cells/well and incubated overnight for 18 h. Cells were then treated with concentrations ranging for 0 to 100 μg/mL of Cbs-Cu or Cbs-Cu-Af for up to 24 h. Post-treatment, MTT assays were performed as detailed in our previous work.^38^

For the spheroid culture, low adherent round-bottom 96 well plates were used. LS174T and HEK-293 cells (1000/well) were added with 200 μL of DMEM with 10% (v/v) FCS along with 2.5% matrigel matrix (Corning, New York). The 96 well plates were then centrifuged for 10 min at 360*g* and then incubated for 48 h for the formation of spheroids. After 48 h, the spheroids were treated with Cbs-Cu-Af for varying time points ranging from 0 to 24 h. Upon completing the treatment period, spheroids were quantified for survivability by incubating with Hoechst 33342 (5 μg/mL) for 30 min and propidium iodide (1.5 μg/mL) for 10 min. Red fluorescent propidium iodide signified the amount of dead cells. Western blots were used to study the fate of cell death by using the apoptosis marker caspase 3.

### *In Vivo* Mice Experiments

Female BALB/c nude mice, aged 6 weeks, each weighing ∼20 g, were used for *in vivo* targeting studies. Mice were sourced from an in-house maintained colony. All experiments were performed following local ethical approval and in accordance with the UK Animals (Scientific Procedures) Act 1986. Mice were housed in individually ventilated cages with a 12 h day/night cycle with provisions for *ad libitum* food and water. At the end of each experiment, mice were euthanized following standard procedures.

CRC xenografts were developed by injecting exponentially growing cells of LS174T (5 × 10^5^ cells), suspended in 100 μL of PBS, subcutaneously in the right flank of the mice. After 10 days tumors were observed, and mice were then randomly divided into separate experiment groups as indicated in each of the experiments.

### *In Vivo* Localization of Cubosomes

The localization of Affimer tagged cubosomes was studied by fluorescence using Cy5 encapsulated cubosomes (Cbs-Cy5). Thirty mice with CRC xenograft were divided into two equal group sizes with 15 animals in each. One of the groups received Cbs-Cy5 (nontargeted) and the second group Cbs-Cy5-Af (Affimer targeted). The administration was done through the tail vein of the mice with 100 μL of sample, equating to 50 mg/kg of cubosome to mouse body weight.

Localization of the Cy5-Cb in the mice was studied by using the Cy5 fluorescence filters in the IVIS Spectrum (PerkinElmer, Inc., Waltham, MA) for a duration of up to 72 h postinjection. At each time point, three mice were euthanized, and the brain, liver, kidney, spleen, heart, and lungs along with the tumor were scanned under the IVIS to quantify the Cy5 fluorescence. The tissues were then frozen in OCT. Sections of 5 μm thickness were made by using a cryostat (Leica CM3050S) and were examined under a confocal microscope (Nikon A1R LSM).

### Efficacy of Targeted Drug Delivery of Affimer Functionalized Cubosomes

Thirty mice bearing CRC xenograft were randomly divided in three groups with 10 mice in each. These groups received the following: saline (control group), nontargeted cubosome with CuAc (Cbs-Cu), and Affimer targeted, drug loaded cubosomes (Cu-Cb-Af). Two doses of 100 μL of intravenous injection containing 25 mg/kg of body weight of cubosome (Cbs-Cu and Cbs-Cu-Af) were administered at day 11 and day 13 (post-tumor inoculation) in the respective group except for the control group which received 100 μL of saline. Tumor volumes were measured by using high-frequency ultrasound (Vevo 770 FUJIFILM Visual Sonics Inc., Toronto, Canada) equipped with a 40 MHz transducer, at regular intervals after the first dose between day 11 and 16.^70^ As per ethical guidelines, mice had to be euthanized as the tumor volume reached the permissible limit of 17 mm diameter. The experiment was terminated on day 16, and all mice were euthanized as most of the control group reached the permissible tumor volume. Post-euthanization, the tumor and other vital organs were collected to study by hematoxylin and eosin staining (i.e., tissue histology). Organs from mice receiving both Cbs-Cu (nontargeted) and Cbs-Cu-Af (targeted group) were homogenized in deionized water (with a resistivity of 18.2 MΩ·cm at 25 °C), and the homogenate was diluted 10-fold. Samples were then subjected to analysis using ICP-OES (iCAP 7600, ICP-OES Analyzer, Thermo Scientific, UK) to estimate the drug uptake, i.e., CuAc content, by using Cu as the reference material. The reference standard for Cu used was provided from the manufacturer (Thermo Scientific, UK).

## Supporting Information Available

Composition and size distribution of cubosomes, encapsulation efficiency of CuAc and stability; characterization of cubosomes using EDAX, FTIR and DLS; characterization of Affimers; carcinoembryonic antigen expression on cells; cytotoxicity of CuAc on cells and mode of action; biocompatibility and selective targeting of cubosome on LS174T cells; *in vivo* cubosome distribution, efficacy on tumor and biosafety (PDF)

## References

[ref1] Mitchell M. J.; Billingsley M. M.; Haley R. M.; Wechsler M. E.; Peppas N. A.; Langer R. Engineering precision nanoparticles for drug delivery. Nat. Rev. Drug Discovery 2021, 20 (2), 101–124. 10.1038/s41573-020-0090-8. 33277608 PMC7717100

[ref2] Barriga H. M. G.; Holme M. N.; Stevens M. M. Cubosomes: The Next Generation of Smart Lipid Nanoparticles?. Angew. Chem., Int. Ed. 2019, 58 (10), 2958–2978. 10.1002/anie.201804067. PMC6606436 29926520

[ref3] Zhai J.; Fong C.; Tran N.; Drummond C. J. Non-Lamellar Lyotropic Liquid Crystalline Lipid Nanoparticles for the Next Generation of Nanomedicine. ACS Nano 2019, 13 (6), 6178–6206. 10.1021/acsnano.8b07961. 31082192

[ref4] Nguyen T.-H.; Hanley T.; Porter C. J. H.; Larson I.; Boyd B. J. Phytantriol and glyceryl monooleate cubic liquid crystalline phases as sustained-release oral drug delivery systems for poorly water-soluble drugs II. In-vivo evaluation. J. Pharm. Pharmacol. 2010, 62 (7), 856–865. 10.1211/jpp.62.07.0006. 20636873

[ref5] Nguyen T.-H.; Hanley T.; Porter C. J. H.; Boyd B. J. Nanostructured liquid crystalline particles provide long duration sustained-release effect for a poorly water soluble drug after oral administration. Journal of controlled release: official journal of the Controlled Release Society 2011, 153 (2), 180–186. 10.1016/j.jconrel.2011.03.033. 21497623

[ref6] Clogston J.; Caffrey M. Controlling release from the lipidic cubic phase. Amino acids, peptides, proteins and nucleic acids. J. Controlled Release 2005, 107 (1), 97–111. 10.1016/j.jconrel.2005.05.015. 15990192

[ref7] Gontsarik M.; Mohammadtaheri M.; Yaghmur A.; Salentinig S. pH-Triggered nanostructural transformations in antimicrobial peptide/oleic acid self-assemblies. Biomater Sci. 2018, 6 (4), 803–812. 10.1039/C7BM00929A. 29383335

[ref8] Negrini R.; Fong W.-K.; Boyd B. J.; Mezzenga R. pH-responsive lyotropic liquid crystals and their potential therapeutic role in cancer treatment. Chem. Commun. 2015, 51 (30), 6671–6674. 10.1039/C4CC10274F. 25783035

[ref9] Zhai J.; Tan F. H.; Luwor R. B.; Srinivasa Reddy T.; Ahmed N.; Drummond C. J.; Tran N. In Vitro and In Vivo Toxicity and Biodistribution of Paclitaxel-Loaded Cubosomes as a Drug Delivery Nanocarrier: A Case Study Using an A431 Skin Cancer Xenograft Model. ACS Applied Bio Materials 2020, 3 (7), 4198–4207. 10.1021/acsabm.0c00269. 35025421

[ref10] Jabłonowska E.; Matyszewska D.; Nazaruk E.; Godlewska M.; Gaweł D.; Bilewicz R. Lipid membranes exposed to dispersions of phytantriol and monoolein cubosomes: Langmuir monolayer and HeLa cell membrane studies. Biochimica et Biophysica Acta (BBA) - General Subjects 2021, 1865 (1), 129738 10.1016/j.bbagen.2020.129738. 32956751

[ref11] Tran N.; Bye N.; Moffat B. A.; Wright D. K.; Cuddihy A.; Hinton T. M.; Hawley A. M.; Reynolds N. P.; Waddington L. J.; Mulet X.; Turnley A. M.; Morganti-Kossmann M. C.; Muir B. W. Dual-modality NIRF-MRI cubosomes and hexosomes: High throughput formulation and in vivo biodistribution. Materials Science and Engineering: C 2017, 71, 584–593. 10.1016/j.msec.2016.10.028. 27987748

[ref12] Zabara A.; Negrini R.; Onaca-Fischer O.; Mezzenga R. Perforated Bicontinuous Cubic Phases with pH-Responsive Topological Channel Interconnectivity. Small 2013, 9 (21), 3602–3609. 10.1002/smll.201300348. 23677679

[ref13] Cortesi R.; Campioni M.; Ravani L.; Drechsler M.; Pinotti M.; Esposito E. Cationic lipid nanosystems as carriers for nucleic acids. New Biotechnology 2014, 31 (1), 44–54. 10.1016/j.nbt.2013.10.001. 24120492

[ref14] Kim H.; Leal C. Cuboplexes: Topologically Active siRNA Delivery. ACS Nano 2015, 9 (10), 10214–10226. 10.1021/acsnano.5b03902. 26390340

[ref15] Janeta M.; Bury W.; Szafert S. Porous Silsesquioxane–Imine Frameworks as Highly Efficient Adsorbents for Cooperative Iodine Capture. ACS Appl. Mater. Interfaces 2018, 10 (23), 19964–19973. 10.1021/acsami.8b03023. 29788716

[ref16] Jain V.; Swarnakar N. K.; Mishra P. R.; Verma A.; Kaul A.; Mishra A. K.; Jain N. K. Paclitaxel loaded PEGylated gleceryl monooleate based nanoparticulate carriers in chemotherapy. Biomaterials 2012, 33 (29), 7206–7220. 10.1016/j.biomaterials.2012.06.056. 22809646

[ref17] Saber M. M.; Al-mahallawi A. M.; Nassar N. N.; Stork B.; Shouman S. A. Targeting colorectal cancer cell metabolism through development of cisplatin and metformin nano-cubosomes. BMC Cancer 2018, 18 (1), 822 10.1186/s12885-018-4727-5. 30111296 PMC6094476

[ref18] Ha S.; La Y.; Kim K. T. Polymer Cubosomes: Infinite Cubic Mazes and Possibilities. Acc. Chem. Res. 2020, 53 (3), 620–631. 10.1021/acs.accounts.9b00563. 31920073

[ref19] He H.; Rahimi K.; Zhong M.; Mourran A.; Luebke D. R.; Nulwala H. B.; Möller M.; Matyjaszewski K. Cubosomes from hierarchical self-assembly of poly(ionic liquid) block copolymers. Nat. Commun. 2017, 8 (1), 14057 10.1038/ncomms14057. 28091605 PMC5241804

[ref20] Aleandri S.; Bandera D.; Mezzenga R.; Landau E. M. Biotinylated Cubosomes: A Versatile Tool for Active Targeting and Codelivery of Paclitaxel and a Fluorescein-Based Lipid Dye. Langmuir 2015, 31 (46), 12770–12776. 10.1021/acs.langmuir.5b03469. 26513646

[ref21] Caltagirone C.; Falchi A. M.; Lampis S.; Lippolis V.; Meli V.; Monduzzi M.; Prodi L.; Schmidt J.; Sgarzi M.; Talmon Y.; Bizzarri R.; Murgia S. Cancer-Cell-Targeted Theranostic Cubosomes. Langmuir 2014, 30 (21), 6228–6236. 10.1021/la501332u. 24815031

[ref22] Zhai J.; Luwor R. B.; Ahmed N.; Escalona R.; Tan F. H.; Fong C.; Ratcliffe J.; Scoble J. A.; Drummond C. J.; Tran N. Paclitaxel-Loaded Self-Assembled Lipid Nanoparticles as Targeted Drug Delivery Systems for the Treatment of Aggressive Ovarian Cancer. ACS Appl. Mater. Interfaces 2018, 10 (30), 25174–25185. 10.1021/acsami.8b08125. 29963859

[ref23] Alcaraz N.; Liu Q.; Hanssen E.; Johnston A.; Boyd B. J. Clickable Cubosomes for Antibody-Free Drug Targeting and Imaging Applications. Bioconjugate Chem. 2018, 29 (1), 149–157. 10.1021/acs.bioconjchem.7b00659. 29182866

[ref24] Sagnella S. M.; Gong X.; Moghaddam M. J.; Conn C. E.; Kimpton K.; Waddington L. J.; Krodkiewska I.; Drummond C. J. Nanostructured nanoparticles of self-assembled lipid pro-drugs as a route to improved chemotherapeutic agents. Nanoscale 2011, 3 (3), 919–924. 10.1039/C0NR00781A. 21173998

[ref25] Biffi S.; Andolfi L.; Caltagirone C.; Garrovo C.; Falchi A. M.; Lippolis V.; Lorenzon A.; Macor P.; Meli V.; Monduzzi M.; Obiols-Rabasa M.; Petrizza L.; Prodi L.; Rosa A.; Schmidt J.; Talmon Y.; Murgia S. Cubosomes forin vivofluorescence lifetime imaging. Nanotechnology 2017, 28 (5), 055102 10.1088/1361-6528/28/5/055102. 28032617

[ref26] Xu X.; Zhang X.; Wei C.; Zheng D.; Lu X.; Yang Y.; Luo A.; Zhang K.; Duan X.; Wang Y. Targeting SLC7A11 specifically suppresses the progression of colorectal cancer stem cells via inducing ferroptosis. European Journal of Pharmaceutical Sciences 2020, 152, 105450 10.1016/j.ejps.2020.105450. 32621966

[ref27] Tiernan J. P.; Perry S. L.; Verghese E. T.; West N. P.; Yeluri S.; Jayne D. G.; Hughes T. A. Carcinoembryonic antigen is the preferred biomarker for in vivo colorectal cancer targeting. British journal of cancer 2013, 108 (3), 662–667. 10.1038/bjc.2012.605. 23322207 PMC3593555

[ref28] Tiernan J. P.; Ingram N.; Marston G.; Perry S. L.; Rushworth J. V.; Coletta P. L.; Millner P. A.; Jayne D. G.; Hughes T. A. CEA-targeted nanoparticles allow specific in vivo fluorescent imaging of colorectal cancer models. Nanomedicine (London) 2015, 10 (8), 1223–1231. 10.2217/nnm.14.202. 25694062

[ref29] Ruigrok V. J. B.; Levisson M.; Eppink M. H. M.; Smidt H.; van der Oost J. Alternative affinity tools: more attractive than antibodies?. Biochem. J. 2011, 436 (1), 1–13. 10.1042/BJ20101860. 21524274

[ref30] Haurum J. S. Recombinant polyclonal antibodies: the next generation of antibody therapeutics?. Drug Discovery Today 2006, 11 (13), 655–660. 10.1016/j.drudis.2006.05.009. 16793535

[ref31] Kyle S. Affimer Proteins: Theranostics of the Future?. Trends Biochem. Sci. 2018, 43 (4), 230–232. 10.1016/j.tibs.2018.03.001. 29550243

[ref32] Tiede C.; Tang A. A. S.; Deacon S. E.; Mandal U.; Nettleship J. E.; Owen R. L.; George S. E.; Harrison D. J.; Owens R. J.; Tomlinson D. C.; McPherson M. J. Adhiron: a stable and versatile peptide display scaffold for molecular recognition applications. Protein Eng. Des. Sel 2014, 27 (5), 145–155. 10.1093/protein/gzu007. 24668773 PMC4000234

[ref33] Tiede C.; Bedford R.; Heseltine S. J.; Smith G.; Wijetunga I.; Ross R.; AlQallaf D.; Roberts A. P.; Balls A.; Curd A.; Hughes R. E.; Martin H.; Needham S. R.; Zanetti-Domingues L. C.; Sadigh Y.; Peacock T. P.; Tang A. A.; Gibson N.; Kyle H.; Platt G. W.; Ingram N.; Taylor T.; Coletta L. P.; Manfield I.; Knowles M.; Bell S.; Esteves F.; Maqbool A.; Prasad R. K.; Drinkhill M.; Bon R. S.; Patel V.; Goodchild S. A.; Martin-Fernandez M.; Owens R. J.; Nettleship J. E.; Webb M. E.; Harrison M.; Lippiat J. D.; Ponnambalam S.; Peckham M.; Smith A.; Ferrigno P. K.; Johnson M.; McPherson M. J.; Tomlinson D. C. Affimer proteins are versatile and renewable affinity reagents. Elife 2017, 10.7554/eLife.24903. PMC5487212 28654419

[ref34] Michel M. A.; Swatek K. N.; Hospenthal M. K.; Komander D. Ubiquitin Linkage-Specific Affimers Reveal Insights into K6-Linked Ubiquitin Signaling. Mol. Cell 2017, 68 (1), 233 10.1016/j.molcel.2017.08.020. 28943312 PMC5640506

[ref35] Shamsuddin S. H.; Jayne D. G.; Tomlinson D. C.; McPherson M. J.; Millner P. A. Selection and characterisation of Affimers specific for CEA recognition. Sci. Rep. 2021, 11 (1), 744 10.1038/s41598-020-80354-6. 33436840 PMC7804248

[ref36] Shamsuddin S. H.; Gibson T. D.; Tomlinson D. C.; McPherson M. J.; Jayne D. G.; Millner P. A. Reagentless Affimer- and antibody-based impedimetric biosensors for CEA-detection using a novel non-conducting polymer. Biosens. Bioelectron. 2021, 178, 113013 10.1016/j.bios.2021.113013. 33508539

[ref37] Krasnovskaya O.; Naumov A.; Guk D.; Gorelkin P.; Erofeev A.; Beloglazkina E.; Majouga A. Copper Coordination Compounds as Biologically Active Agents. Int. J. Mol. Sci. 2020, 21 (11), 3965 10.3390/ijms21113965. PMC7312030 32486510

[ref38] Pramanik A.; Laha D.; Dash S. K.; Chattopadhyay S.; Roy S.; Das D. K.; Pramanik P.; Karmakar P. An in-vivo study for targeted delivery of copper-organic complex to breast cancer using chitosan polymer nanoparticles. Materials Science and Engineering: C 2016, 68, 327–337. 10.1016/j.msec.2016.05.014. 27524027

[ref39] Pramanik A.; Laha D.; Pramanik P.; Karmakar P. A novel drug “copper acetylacetonate” loaded in folic acid-tagged chitosan nanoparticle for efficient cancer cell targeting. J. Drug Targeting 2014, 22, 23 10.3109/1061186X.2013.832768. 23987131

[ref40] Jia Y.-Y.; Zhang J.-J.; Zhang Y.-X.; Wang W.; Li C.; Zhou S.-Y.; Zhang B.-L. Construction of redox-responsive tumor targeted cisplatin nano-delivery system for effective cancer chemotherapy. Int. J. Pharm. 2020, 580, 119190 10.1016/j.ijpharm.2020.119190. 32151664

[ref41] Peng J. Q.; Fumoto S.; Suga T.; Miyamoto H.; Kuroda N.; Kawakami S.; Nishida K. Targeted co-delivery of protein and drug to a tumor in vivo by sophisticated RGD-modified lipid-calcium carbonate nanoparticles. J. Controlled Release 2019, 302, 42–53. 10.1016/j.jconrel.2019.03.021. 30926479

[ref42] Sykes E. A.; Chen J.; Zheng G.; Chan W. C. W. Investigating the Impact of Nanoparticle Size on Active and Passive Tumor Targeting Efficiency. ACS Nano 2014, 8 (6), 5696–5706. 10.1021/nn500299p. 24821383

[ref43] Tang L.; Yang X.; Yin Q.; Cai K.; Wang H.; Chaudhury I.; Yao C.; Zhou Q.; Kwon M.; Hartman J. A.; Dobrucki I. T.; Dobrucki L. W.; Borst L. B.; Lezmi S.; Helferich W. G.; Ferguson A. L.; Fan T. M.; Cheng J. Investigating the optimal size of anticancer nanomedicine. Proc. Natl. Acad. Sci. U. S. A. 2014, 111 (43), 15344 10.1073/pnas.1411499111. 25316794 PMC4217425

[ref44] Tan C.-P.; Lu Y.-Y.; Ji L.-N.; Mao Z.-W. Metallomics insights into the programmed cell death induced by metal-based anticancer compounds. Metallomics 2014, 6 (5), 978–995. 10.1039/c3mt00225j. 24668273

[ref45] Bazylińska U.; Kulbacka J.; Schmidt J.; Talmon Y.; Murgia S. Polymer-free cubosomes for simultaneous bioimaging and photodynamic action of photosensitizers in melanoma skin cancer cells. J. Colloid Interface Sci. 2018, 522, 163–173. 10.1016/j.jcis.2018.03.063. 29601958

[ref46] Kulkarni C. V.; Tang T.-Y.; Seddon A. M.; Seddon J. M.; Ces O.; Templer R. H. Engineering bicontinuous cubic structures at the nanoscale—the role of chain splay. Soft Matter 2010, 6 (14), 3191–3194. 10.1039/c0sm00068j.

[ref47] Briggs J.; Chung H.; Caffrey M. The Temperature-Composition Phase Diagram and Mesophase Structure Characterization of the Monoolein/Water System. J. Phys. II France 1996, 6 (5), 723–751. 10.1051/jp2:1996208.

[ref48] Barauskas J.; Johnsson M.; Joabsson F.; Tiberg F. Cubic Phase Nanoparticles (Cubosome): Principles for Controlling Size, Structure, and Stability. Langmuir 2005, 21 (6), 2569–2577. 10.1021/la047590p. 15752054

[ref49] Sagalowicz L.; Michel M.; Adrian M.; Frossard P.; Rouvet M.; Watzke H. J.; Yaghmur A.; De Campo L.; Glatter O.; Leser M. E. Crystallography of dispersed liquid crystalline phases studied by cryo-transmission electron microscopy. J. Microsc. 2006, 221 (2), 110–121. 10.1111/j.1365-2818.2006.01544.x. 16499550

[ref50] Grunnet M.; Sorensen J. B. Carcinoembryonic antigen (CEA) as tumor marker in lung cancer. Lung Cancer 2012, 76 (2), 138–143. 10.1016/j.lungcan.2011.11.012. 22153832

[ref51] Kaushal S.; McElroy M. K.; Luiken G. A.; Talamini M. A.; Moossa A. R.; Hoffman R. M.; Bouvet M. Fluorophore-conjugated anti-CEA antibody for the intraoperative imaging of pancreatic and colorectal cancer. J. Gastrointest Surg 2008, 12 (11), 1938–1950. 10.1007/s11605-008-0581-0. 18665430 PMC4396596

[ref52] Lázaro-Gorines R.; Ruiz-de-la-Herrán J.; Navarro R.; Sanz L.; Álvarez-Vallina L.; Martínez-del-Pozo A.; Gavilanes J. G.; Lacadena J. A novel Carcinoembryonic Antigen (CEA)-Targeted Trimeric Immunotoxin shows significantly enhanced Antitumor Activity in Human Colorectal Cancer Xenografts. Sci. Rep. 2019, 9 (1), 11680 10.1038/s41598-019-48285-z. 31406218 PMC6690998

[ref53] Hama S.; Itakura S.; Nakai M.; Nakayama K.; Morimoto S.; Suzuki S.; Kogure K. Overcoming the polyethylene glycol dilemma via pathological environment-sensitive change of the surface property of nanoparticles for cellular entry. J. Controlled Release 2015, 206, 67–74. 10.1016/j.jconrel.2015.03.011. 25770398

[ref54] Lee J. M.; Choi J. W.; Ahrberg C. D.; Choi H. W.; Ha J. H.; Mun S. G.; Mo S. J.; Chung B. G. Generation of tumor spheroids using a droplet-based microfluidic device for photothermal therapy. Microsystems & Nanoengineering 2020, 6 (1), 52 10.1038/s41378-020-0167-x. 34567663 PMC8433304

[ref55] Costa E. C.; Moreira A. F.; de Melo-Diogo D.; Gaspar V. M.; Carvalho M. P.; Correia I. J. 3D tumor spheroids: an overview on the tools and techniques used for their analysis. Biotechnology Advances 2016, 34 (8), 1427–1441. 10.1016/j.biotechadv.2016.11.002. 27845258

[ref56] Loessner D.; Stok K. S.; Lutolf M. P.; Hutmacher D. W.; Clements J. A.; Rizzi S. C. Bioengineered 3D platform to explore cell–ECM interactions and drug resistance of epithelial ovarian cancer cells. Biomaterials 2010, 31 (32), 8494–8506. 10.1016/j.biomaterials.2010.07.064. 20709389

[ref57] Ireson C. R.; Alavijeh M. S.; Palmer A. M.; Fowler E. R.; Jones H. J. The role of mouse tumour models in the discovery and development of anticancer drugs. Br. J. Cancer 2019, 121 (2), 101–108. 10.1038/s41416-019-0495-5. 31231121 PMC6738037

[ref58] Prange J. A.; Aleandri S.; Komisarski M.; Luciani A.; Käch A.; Schuh C.-D.; Hall A. M.; Mezzenga R.; Devuyst O.; Landau E. M. Overcoming Endocytosis Deficiency by Cubosome Nanocarriers. ACS Applied Bio Materials 2019, 2 (6), 2490–2499. 10.1021/acsabm.9b00187. 35030705

[ref59] Haute D. V.; Berlin J. M. Challenges in realizing selectivity for nanoparticle biodistribution and clearance: lessons from gold nanoparticles. Therapeutic Delivery 2017, 8 (9), 763–774. 10.4155/tde-2017-0057. 28825391 PMC6123877

[ref60] De Jong W. H.; Hagens W. I.; Krystek P.; Burger M. C.; Sips A. J. A. M.; Geertsma R. E. Particle size-dependent organ distribution of gold nanoparticles after intravenous administration. Biomaterials 2008, 29 (12), 1912–1919. 10.1016/j.biomaterials.2007.12.037. 18242692

[ref61] Li S.; Zhang J.; Deng C.; Meng F.; Yu L.; Zhong Z. Redox-Sensitive and Intrinsically Fluorescent Photoclick Hyaluronic Acid Nanogels for Traceable and Targeted Delivery of Cytochrome c to Breast Tumor in Mice. ACS Appl. Mater. Interfaces 2016, 8 (33), 21155–21162. 10.1021/acsami.6b05775. 27509045

[ref62] Izci M.; Maksoudian C.; Manshian B. B.; Soenen S. J. The Use of Alternative Strategies for Enhanced Nanoparticle Delivery to Solid Tumors. Chem. Rev. 2021, 121 (3), 1746–1803. 10.1021/acs.chemrev.0c00779. 33445874 PMC7883342

[ref63] Feng H.-Y.; Yuan Y.; Zhang Y.; Liu H.-J.; Dong X.; Yang S.-C.; Liu X.-L.; Lai X.; Zhu M.-H.; Wang J.; Lu Q.; Lin Q.; Chen H.-Z.; Lovell J. F.; Sun P.; Fang C. Targeted Micellar Phthalocyanine for Lymph Node Metastasis Homing and Photothermal Therapy in an Orthotopic Colorectal Tumor Model. Nano-Micro Letters 2021, 13 (1), 145 10.1007/s40820-021-00666-8. 34146159 PMC8214644

[ref64] Wei Y.; Gu X.; Sun Y.; Meng F.; Storm G.; Zhong Z. Transferrin-binding peptide functionalized polymersomes mediate targeted doxorubicin delivery to colorectal cancer in vivo. Journal of controlled release: official journal of the Controlled Release Society 2020, 319, 407–415. 10.1016/j.jconrel.2020.01.012. 31923538

[ref65] Esteban-Fernández D.; Verdaguer J. M.; Ramírez-Camacho R.; Palacios M. A.; Gómez-Gómez M. M. Accumulation, Fractionation, and Analysis of Platinum in Toxicologically Affected Tissues after Cisplatin, Oxaliplatin, and Carboplatin Administration. Journal of Analytical Toxicology 2008, 32 (2), 140–146. 10.1093/jat/32.2.140. 18334097

[ref66] Wei Y.; Gu X.; Sun Y.; Meng F.; Storm G.; Zhong Z. Transferrin-binding peptide functionalized polymersomes mediate targeted doxorubicin delivery to colorectal cancer in vivo. J. Controlled Release 2020, 319, 407–415. 10.1016/j.jconrel.2020.01.012. 31923538

[ref67] Basham M.; Filik J.; Wharmby M. T.; et al. Data Analysis WorkbeNch (DAWN). Journal of Synchrotron Radiation 2015, 22 (3), 853–858. 10.1107/S1600577515002283. 25931106 PMC4416692

[ref68] Filik J.; Ashton A. W.; Chang P. C. Y.; Chater P. A.; Day S. J.; Drakopoulos M.; Gerring M. W.; Hart M. L.; Magdysyuk O. V.; Michalik S.; Smith A.; Tang C. C.; Terrill N. J.; Wharmby M. T.; Wilhelm H. Processing two-dimensional X-ray diffraction and small-angle scattering data in DAWN 2. J. Appl. Crystallogr. 2017, 50 (3), 959–966. 10.1107/S1600576717004708. 28656043 PMC5458597

[ref69] Tinevez J.-Y.; Perry N.; Schindelin J.; Hoopes G. M.; Reynolds G. D.; Laplantine E.; Bednarek S. Y.; Shorte S. L.; Eliceiri K. W. TrackMate: An open and extensible platform for single-particle tracking. Methods 2017, 115, 80–90. 10.1016/j.ymeth.2016.09.016. 27713081

[ref70] Ingram N.; Macnab S. A.; Marston G.; Scott N.; Carr I. M.; Markham A. F.; Whitehouse A.; Coletta P. L. The use of high-frequency ultrasound imaging and biofluorescence for in vivo evaluation of gene therapy vectors. BMC Med. Imaging 2013, 13, 35 10.1186/1471-2342-13-35. 24219244 PMC3831818

