# Supplementary material for: Affimer Tagged Cubosomes: Targeting of Carcinoembryonic Antigen Expressing Colorectal Cancer Cells Using In Vitro and In Vivo Models
Source: ACS Appl Mater Interfaces. 2022 Feb 23;14(9):11078–91. doi: 10.1021/acsami.1c21655 (PMC9007418; doi:10.1021/acsami.1c21655)
Supplement: Supplementary file 1 — am1c21655_si_001.pdf [file am1c21655_si_001.pdf]

Supporting Information

Affimer Tagged Cubosomes: Targeting of Carcinoembryonic Antigen Expressing Colorectal Cancer Cells using *In Vitro* and *In Vivo* Models

Arindam Pramanik[1,2], Zexi Xu[3,4], Shazana H. Shamsuddin[1,5], Yazan S. Khaled[2], Nicola Ingram[6], Thomas Maisey[2], Darren Tomlinson[7],P. Louise Coletta[6], David Jayne[6], Thomas A. Hughes[2*], Arwen I.I. Tyler[3*] and Paul A. Millner[1*]*

[1]School of Biomedical Sciences, University of Leeds, Leeds, LS2 9JT, United Kingdom

[2]School of Medicine, University of Leeds, Leeds, LS9 7TF, United Kingdom

[3]School of Food Science and Nutrition, University of Leeds, Leeds, LS2 9JT, United Kingdom

[4]School of Chemistry and Astbury Centre for Structural Molecular Biology, University of Leeds, Leeds, LS2 9JT, United Kingdom

[5]Department of Pathology, School of Medical Sciences, Universiti Sains Malaysia, 16150, Malaysia

[6]Leeds Institute of Medical Research, St James's University Hospital, Leeds, LS9 7TF, United Kingdom

[7]Biomedical Health Research Centre, BioScreening Technology Group, University of Leeds, Leeds, LS2 9JT, United Kingdom

Corresponding Authors

* Emails: A.Pramanik@leeds.ac.uk; T.Hughes@leeds.ac.uk; A.I.I.Tyler@leeds.ac.uk; P.A.Millner@leeds.ac.uk

Material & Methods

Cytotoxicity of copper acetyacetonate

LS174T and HEK-293 cells were seeded in 24 well culture plates in DMEM at cell densities of 2.5 x 10^4 cells/well and incubated overnight for 18 h. Cells were then treated with 0 – 25 µg/ml of CuAc (Merck, New Jersey, USA) dissolved in DMSO (Merck, New Jersey, USA) where the maximum concentration of DMSO in the media was kept below 1%. After 24 h, cells were incubated with 0.5 mg/ml of MTT dissolved in PBS for 3 h. The formazan crystals formed within the cells were dissolved using 500 µl of isopropanol and the absorbance was measured at 570nm on a multiwell plate reader (Biotech Instruments, USA). Similarly, spheroids of HEK-293 and LS174T after 48h of incubation were treated with 0 – 60 µg/ml of CuAc for 24h and then survivability was accessed using Hoechst 33342 (5µg/ml) for 30mins and propidium iodide (1.5 µg/ml) for 10mins.

Analysis of reactive oxygen species

The effect of CuAc on the intracellular ROS levels was estimated using 2,7-dichlorofluorescein diacetate (DCF-DA,Merck, New Jersey, USA). Briefly, cells were treated with the respective LD_{50} of CuAc for up to 24 h then incubated with 10 µM DCF-DA in Hank's balanced salt solution (Merck, New Jersey, USA) for 30 min. Cells were washed with PBS and observed under a fluorescent microscope (EVOS FL auto, Thermo fisher Scientific, UK). A separate set of cells were trypsinized (post CuAc treatment), washed with 1x PBS, then stained with 5 µM DCF-DA in Hank's balanced salt solution for 30 min and analyzed under a spectro-fluorimeter (RF-6000 Shimadzu, UK) with excitation/emission wavelengths of 488 nm/525 nm respectively

Measurement of mitochondrial membrane potential

JC-1 is a membrane permeant dye used to monitor the mitochondria membrane potential. Upon treatment of HEK-293 and LS174T cells with respective LD_{50} of CuAc for 12 h and 24 h, cells were incubated with 5 µM JC-1 dye (Thermo fisher Scientific, Waltham, MA, USA) for 30min at 37ºC. Then cells were analyzed under a fluorescence microscope (EVOS FL auto, Thermo fisher Scientific, UK) with an excitation wavelength of 488 nm and an emission range between 515/545 nm and 575/625 nm respectively.

Additionally, to analyse mitochondrial potential, a separate set of cells were trypsinized after LD_{50} CuAc treatment at 12 h and 24 h and then incubated with 2 µM JC-1 for 15 min at 37°C, 5% CO_2. Cells were then washed with PBS and analyzed on a CytoFLEX S flow cytometer (Beckman, California, USA). The data was analyzed using the FlowJo software (version 10.6.1).

Annexin V apoptosis assay by flow cytometry

After treatment of cells with respective LD_{50} of CuAc at different time intervals, cells were trypsinized and washed with ice cold PBS and suspended in annexin binding buffer (Thermo fisher Scientific, Waltham, MA, USA). Annexin V- FITC (Thermo fisher Scientific, Waltham, MA, USA) at a final concentration of 2 µg/ml was added to the cells and incubated for 15 min under dark conditions. Prior to flow cytometry, 2 µg/ml 7-AAD (Thermo fisher Scientific, Waltham, MA, USA) was added and cells were analyzed using a CytoFLEXS flow cytometer. The data was analyzed using the FlowJo software (version 10.6.1).

Western blot assay for protein expression

Cells were harvested with or without respective treatment conditions and washed with ice-cold PBS. Then, cells were incubated for 45 min in an ice bath with RIPA lysis buffer (Thermo Scientific, USA) with agitation at regular intervals to isolate whole cell protein extract. The lysate was then collected by centrifuging at 13,000 rpm for 10 min to remove any cell debris. The protein concentration was analyzed using a BCA reagent (Merck, New Jersey, USA) against a known standard curve. The protein lysate was mixed with 2X Laemmle sample loading buffer (Thermo Scientific, USA) and heated for 5min at 90ºC. Gel electrophoresis was performed for 90 min at 120V on a 4 - 12% precast polyacrylamide gel (Bio Rad, California, USA). The proteins were then transferred to a PVDF membrane and blocked with 5% (w/v) non-fat skimmed milk in TBST (Tris buffered saline with 0.1% Tween-20) for 1h. The membrane was further incubated with respective primary antibodies of anti-CEA (Thermo Scientific, USA), caspase 3 and 9, PARP and β-actin (Cell Signalling Technologies, Beverly, USA). Antibody dilution used was in range from 1:1000 - 1:1500 in TBST, overnight at 4ºC. Next, the membrane was washed with TBST for 1h and then labeled with respective HRP tagged secondary antibody (Cell Signalling Technologies, Beverly, USA) at a 1:5000 ratio in TBST, for 2 h at room temperature. The protein bands on the membrane were visualized by adding Pierce™ ECL reagent and quantified using a chemi-doc instrument

(Biorad, USA). Band intensities were measured from independent replicates using ImageJ (NIH, USA).

Drug release experiments

The drug release study was done using the reverse dialysis method.[1] 10mg/ml c of Cbs-Cu-Af in 1X PBS was placed under stirring conditions at 37ºC. A Slide-A-Lyzer™ MINI dialysis tube of 2K MWCO (Thermo Scientific USA) was placed in the PBS solution to entrap the free CuAc released from the cubosome. At regular intervals,100µl of the samples were taken from the dialysis tube (same amount of 1X PBS replaced) and was used for analysis of copper content using ICP-OES (similar to the report used by Nguyen *et al.* for detection of cisplatin).[2] Similarly, a known concentration of CuAc was stirred placed in a dialysis tube was used to check for release kinetics for free drug.

Biophysical characterization of cubosomes and encapsulation efficiency

MO: DPA (w/w) %	F127 (w/w % of MO & DPA)	Average size (nm)	PDI
90 : 10	5	296	0.31
90: 10	7	220	0.43
95: 5	2	202	0.26

Table S1. Cubosome synthesis and size distribution study by DLS. The hydrodynamic diameter of cubosomes and their polydispersity index (PDI) with different ratio of MO, DPA and pluronic F127 as stabilizer. Data shows 95: 5 w/w % of MO:DPA and 7% (w/w) F127 produced the smallest size (106 nm) of bare cubosomes and thus this composition was selected for further studies.

95 : 5	5	186	0.21
95 : 5	7	106	0.115

Table S2. Encapsulation efficiency of CuAc in cubosomes with different loading percentage as obtained from ICP-OES. [a]The total weight was obtained by taking 95:5% (MO:DPA) and 7% (w/w of MO and DPA) of F127 i.e., 55mg of MO, 2.89mg DPA and 4.05 mg F127. [b]The weight of CuAc was calculated based on formula weight, as ICP-OES provided the weight of Cu in the sample. 5% (w/w) of CuAc (with respect to MO)was found to have 82% encapsulation efficiency and was taken forward for subsequent studies. All these samples were stable for more than 21days. Further increase of drug loading resulted in unstable samples.

Total Weight of MO, DPA & F127 (mg)[a]	Weight of CuAc added (mg)	Weight of CuAc derived from ICP-OES (mg)[b]	Encapsulation Efficiency (%)	Stability (days)
61.94	0.57	0.43	76	>21
61.94	1.44	1.25	87	>21
61.94	2.89	2.37	82	>21

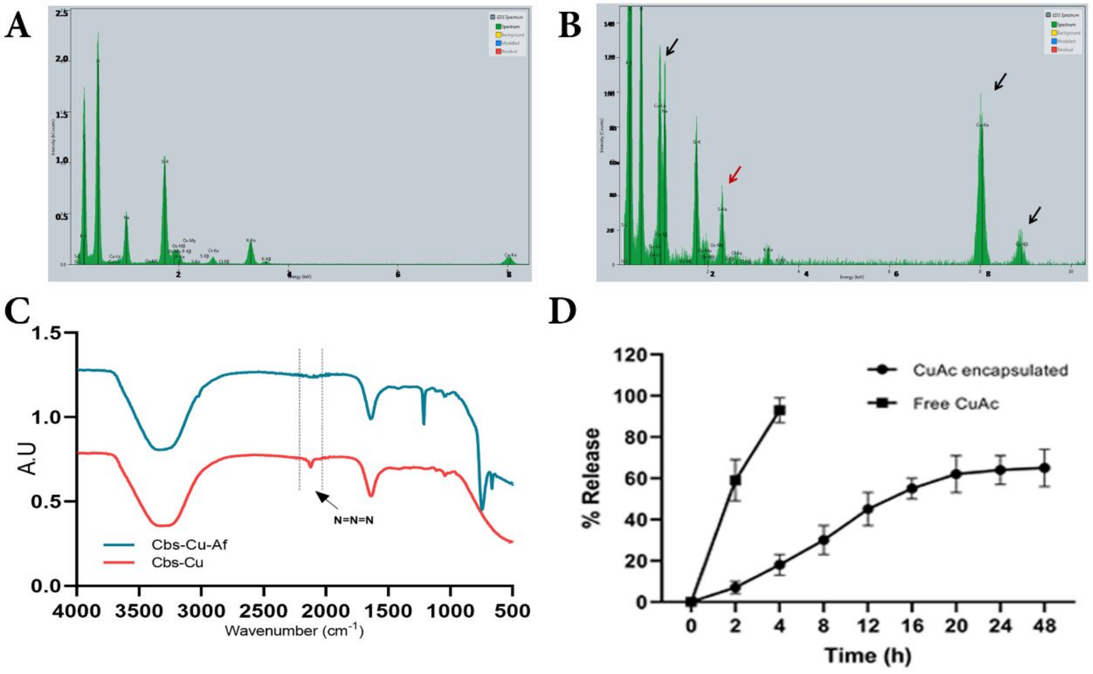

Figure S1: Chemical characterization of cubosome upon functionalization and drug loading. Energy Dispersive X-Ray analysis (EDAX) of **(A)** bare Cbs and (**B**) CuAc encapsulated cubosomes tagged with Affimer (Cbs-Cu-Af). The latter showing distinct peaks for Copper (black arrows) which confirms the presence of CuAc in the cubosome. The characteristic Sulphur Kα and Kβ peaks (2.3 – 2.5 eV) arising from the tagged cubosomes are also seen here (red arrows) that is due to cysteine present in the Affimer **(C)** Fourier-transform infrared spectra of Affimer tagged (Cbs-Cu-Af) and untagged (Cbs-Cu) cubosomes loaded with CuAc show that Cbs-Cu shows a peak at 2127 cm^{-1} attributed to the azide (N=N=N) functional group . After conjugation of DBCO Affimer to the DSPE-azide group on the cubosomes, the peak is absent in the spectrum for Cbs-Cu-Af which confirmes the covalent linkage of Affimer to the cubosomesurface and is not just physical adsoprtion. (D) Drug release data as determined by ICPOES concluded that CuAc has a slow and sustained release from the cubosomes.

A

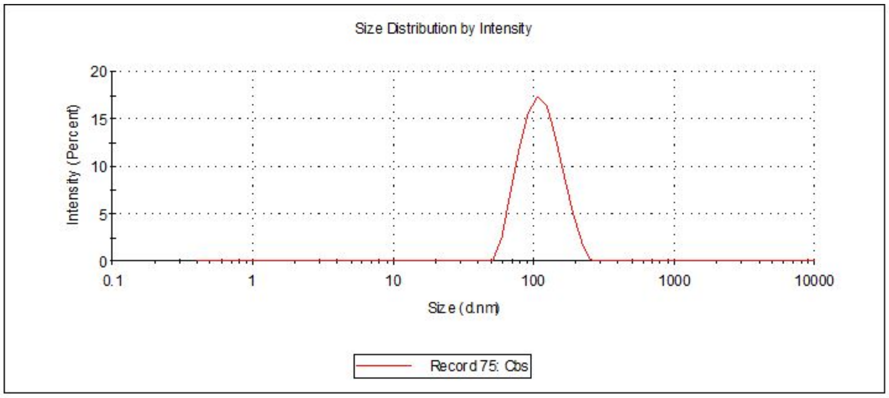

B

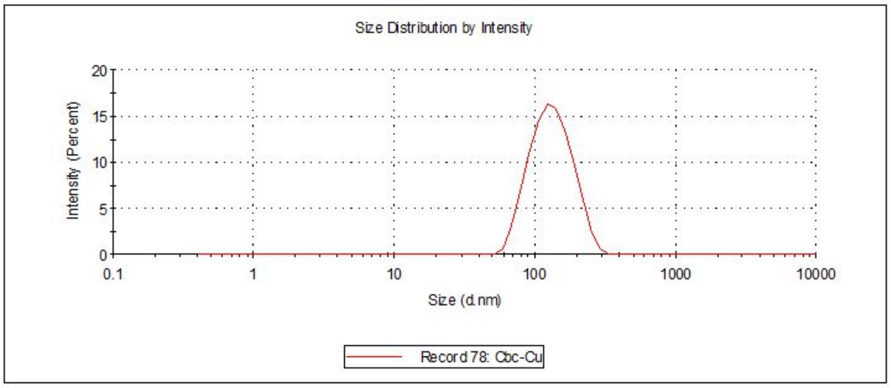

C

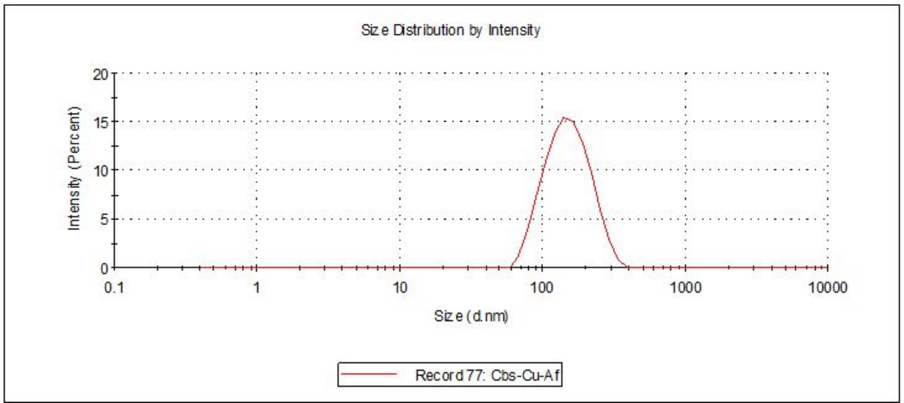

Figure S2: Dynamic light scattering data showing the hydrodynamic diameter of **(A)** bare cubosome (Cbs), **(B)** CuAc encapsulated cubosome (Cbs-Cu) and **(C)** CuAc loaded cubosome tagged with Affimer (Cbs-Cu-Af) showing the z-average size was 106, 121 and 141nm respectively.

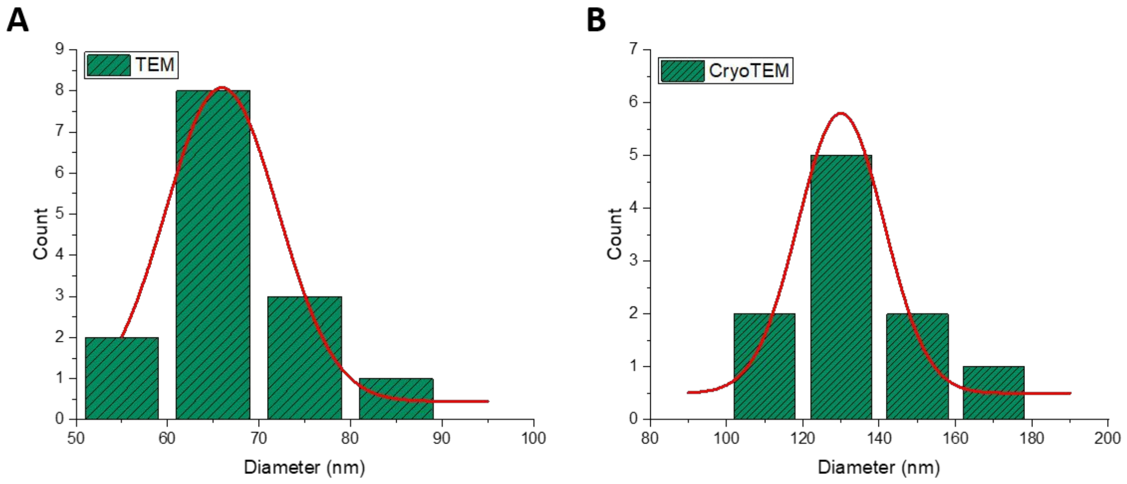

Figure S3. TEM (**A**) and Cryo-TEM (**B**) size distribution analysis of Cbs-Cu-Af cubosomes. The histograms were fitted to a Gaussian function. The mean diameter of Cbs-Cu-Af from TEM was found to be 65.95 nm with a SD of ±14.5, whereas the mean diameter of Cbs-Cu-Af from Cryo-TEM was found to be 130 nm with a SD of ± 26.5.

Affimer proteins as promising ligands for carcinoembryonic antigen on colorectal cancer cells

CEA binding Affimer proteins were identified using a 'phage display library method' by Shamsuddin et al. [3]. Out of the three CEA binding Affimers identified, clone II and III were chosen for this study having 9 and 10 distinct amino acid residues at the variable region in clone II and clone III respectively. Based on a 50 ml of working volume, the yield of the Affimers was 8.3 mg and 6.27 mg for clone II and III respectively[3-4] with corresponding molecular weights noted to be 12.5 and 12.6 kDa **(Figure S3A)**. The surface plasmon resonance showed that the Affimers demonstrated a high binding affinity towards CEA (K_d for clone II:15.3 ± 0.37 nM; Kd for clone III: 34.4 ± 16 nM) **(Figure S3B)**. Interestingly when compared to CEA mAb, these Affimers showed enhanced binding to cell surface CEA as studied from immunofloresence assay on CRC cells.

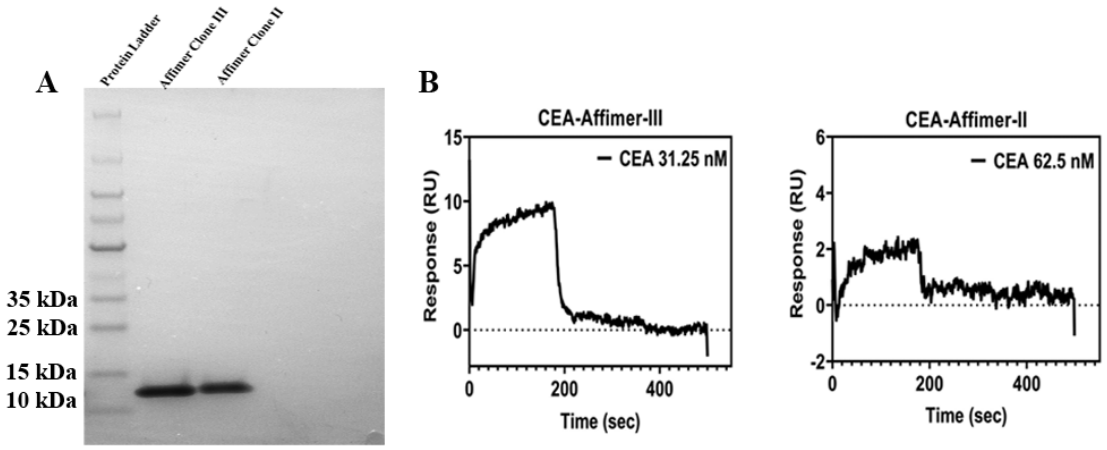

Figure S4: (A) SDS-page gel showing the molecular weight of the CEA-Affimer clone III and clone II along with a standard protein ladder; The Affimer protein was found to have a molecular weight of 12.5 and 12.6 kDa for clone III and clone II respectively. **(B)** Surface plasma resonance (SPR) showing the strong binding Affinity of Affimer clone III and clone II to the CEA antigen for as low as 31.25nM and 62.5nM concentrations respectively.

***In vivo* and *in vitro* studies**

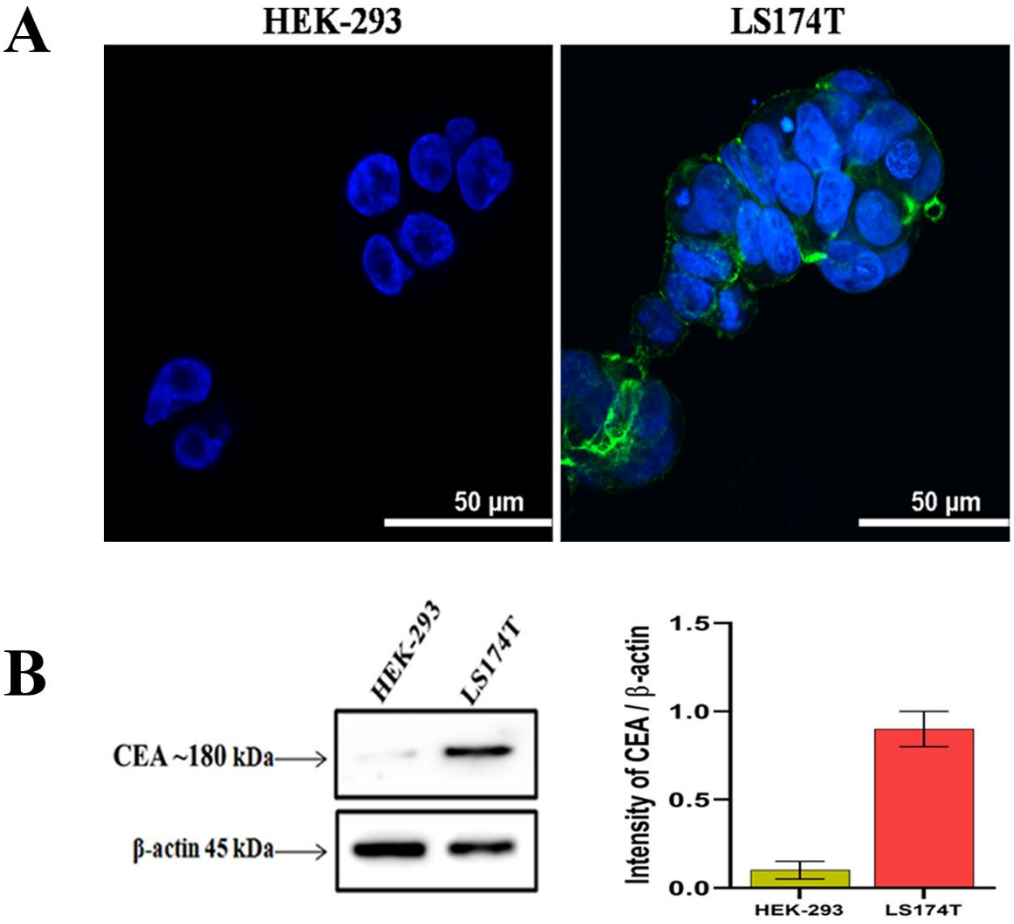

Figure S5: Expression of CEA on LS174T and HEK-293 cells as studied from (A) Immunoflourescence assay by confocal microscopy. CEA expression is indicated by the green fluorescence of anti-CEA antibody labeled with Alexa Fluor 488 secondary antibody whereas blue fluorescence is from the nucleus of cells stained with DAPI. As evident LS174T cells had high expression of CEA whereas CEA expression is absent in HEK-293 cells. This was further validated by (B) western blots along with their quantitative band intensities, where CEA protein is over-expressed in LS174T up to 9 fold compared to negligible expression in HEK-293 cells.

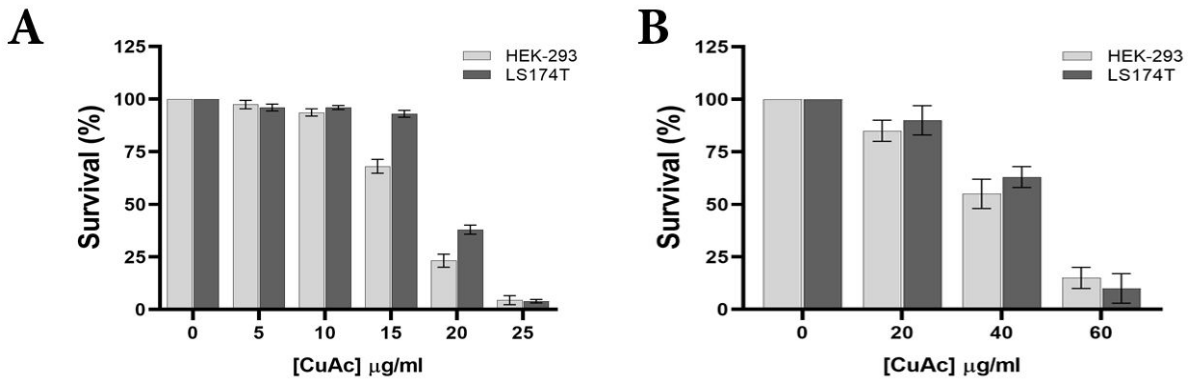

Figure S6: Cytotoxicity study of CuAc on cells; **(A)** 24h Cell cytotoxicity data of CuAc on HEK-293 and LS174T cells at various concentrations between 0- 25 μg/ml. LD_{50} value for HEK-293 was 16 μg/ml and LS174T was 19 μg/ml. **(B)** Cytotoxicity evaluation of CuAc (0- 60 μg/ml) was studied on 3D spheroids of the two cell lines for 24h. The survivability of spheroids was calculated by measuring red fluorescent from propidium iodide indicating dead cells and counter stained with Hoechst 33342 giving blue fluorescence from live cells. Spheroids were slightly more resistant to CuAc and had an LD_{50} value of 40 and 45 μg/ml for HEK-293 and LS174T cells respectively. Conclusive that CuAc had no specificity towards colorectal cancer cells**.**

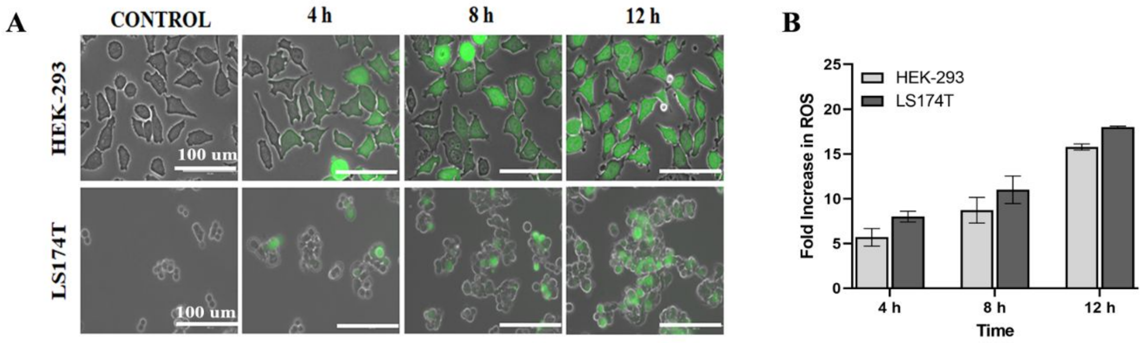

Figure S7: Reactive oxygen species (ROS) generation was observed due to CuAc treatment on cells. **(A)** The intensity of DCF-DA (green) increased in both the cells when treated with CuAc at the respective LD_{50} dose (16 and 19 μg/ml for HEK-293 and LS174T respectively). The increase in green fluorescence intensity denoted increase in intracellular ROS generation after 4h of treatment with CuAc. (B) ROS intensity was measured with a spectroflourimeter at various time points and similar observations were noted.

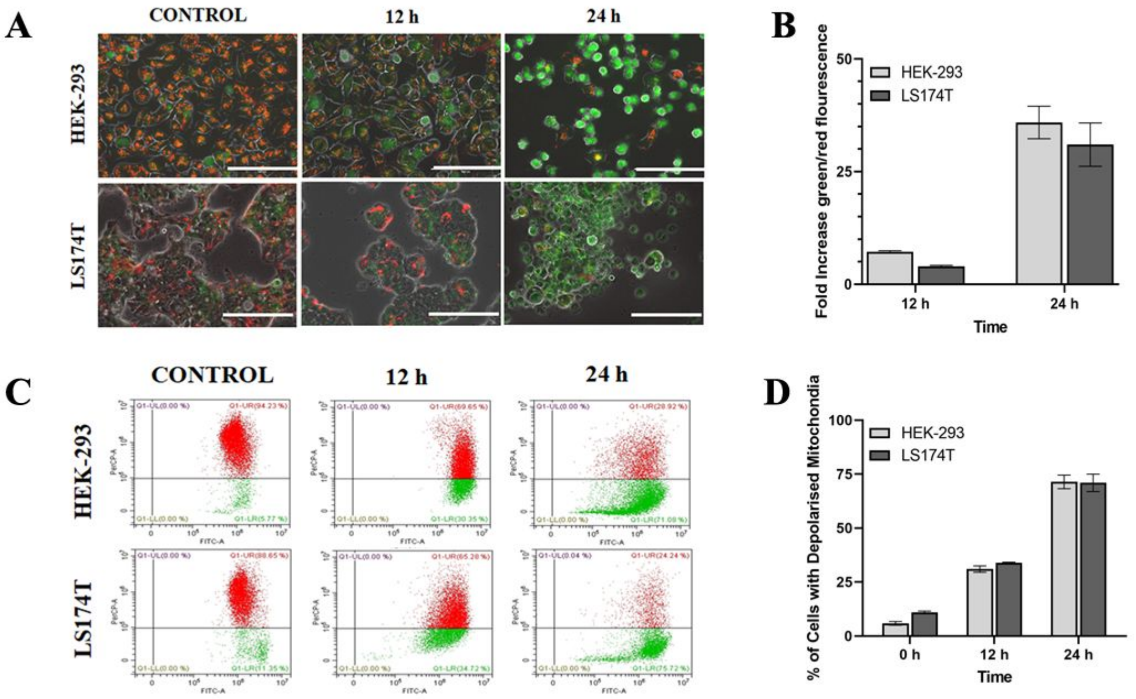

Figure S8: (A) The mitochondrial membrane potential of cells was studied using 5 μM JC-1 dye under a fluorescence microscope upon treatment with the respective LD_{50} of CuAc at 12h and 24h. The shift in fluorescence from red to green indicates depolarization of mitochondria. **(B)** Quantification of green and red fluorescence intensity. The fold increase of green/ red fluorescence post treatment with CuAc confirmed the event of mitochondrial depolarization; **(C)** The treated HEK293 and LS174T cells were analysed in a flow cytometer after incubating with 2 μM JC-1. The cell population was observed to migrate from red towards green after 12h of treatment. **(D)** The quantitative increase in cell population with green fluorescence concluded mitochondria depolarization or membrane damage upon ROS generation.

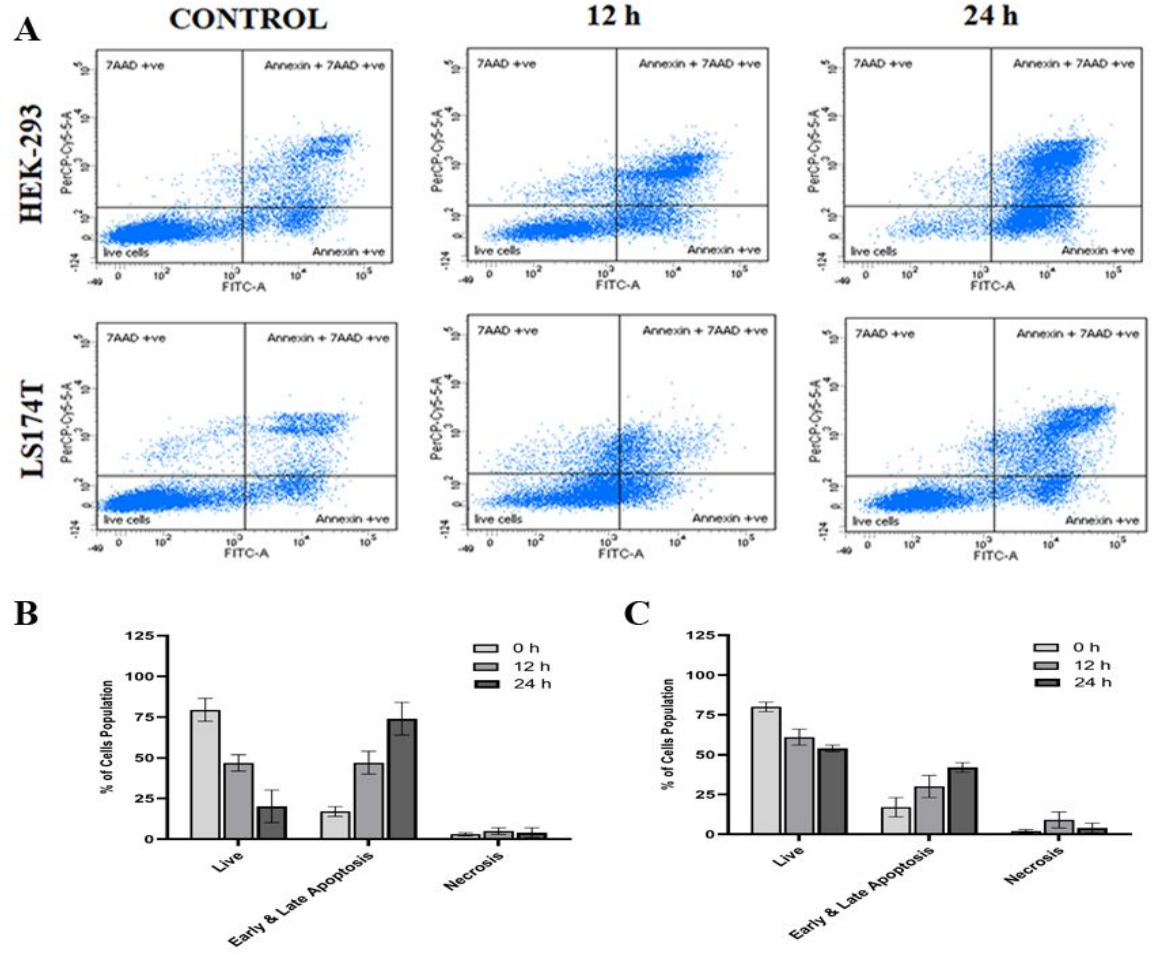

Figure S9: Fate of cell death by flow cytometry. **(A)** Apoptosis was studied after cells were treated with the respective LD_{50} of CuAc up to 24h. From flow cytometery, it was evident that the apoptotic cell population increased after 12h of treatment as indicated by Annexin V positive cells. The quantitative graph shows that with increasing treatment time the percentage of apoptosis in cells increased in both; **(B)** HEK-293 and **(C)** LS174T cells.

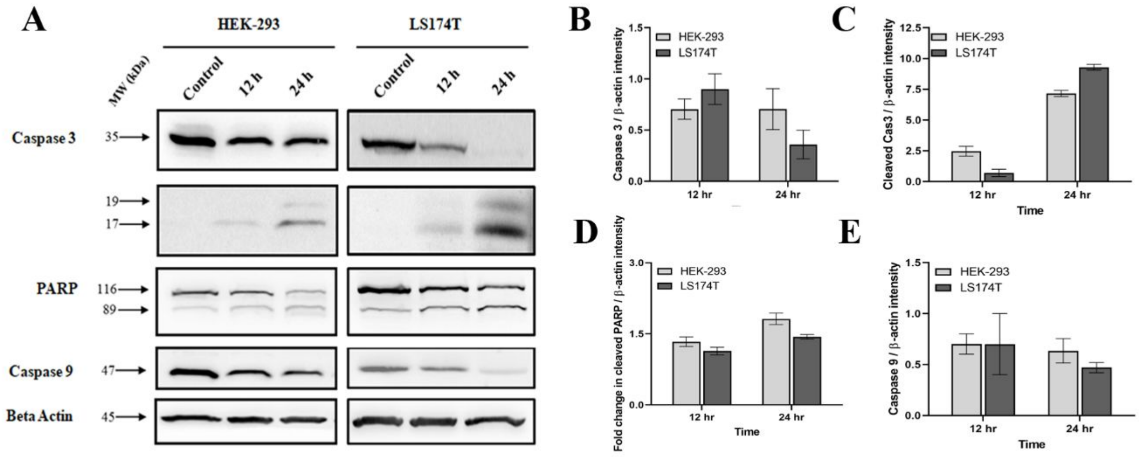

Figure S10: Western blots for apoptotic marker identification. **(A)** Apoptosis was further confirmed from western blots using the apoptotic protein markers caspase 3, caspase 9 and Parp. From the blot intensity plots it is clear that upon treatment of cells with CuAc at LD_{50} **(B)** the full length caspase 3 decreased and **(C)** cleaved caspase 3 increased; **(D)** Parp cleavage increased and **(E)** full length caspase 9 decreased. These observation confirmed that CuAc induced apoptosis mediated cell death in both HEK-293 and LS174T. CuAc shows no specificity towards cell lines.

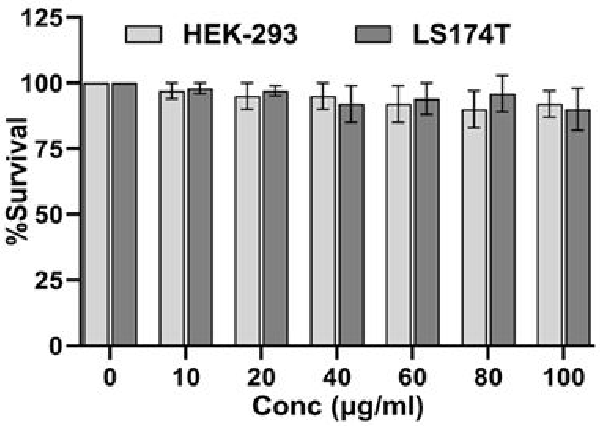

Figure S11: MTT assay of bare cubosome (95:5 % MO: DPA and 7% F127) on HEK-293 and LS174T showed no reduction of cell viability up to 100 µg/ml for 24h concluding the biocompatibility of MO:DPA:F127 cubosomes at these concentrations.

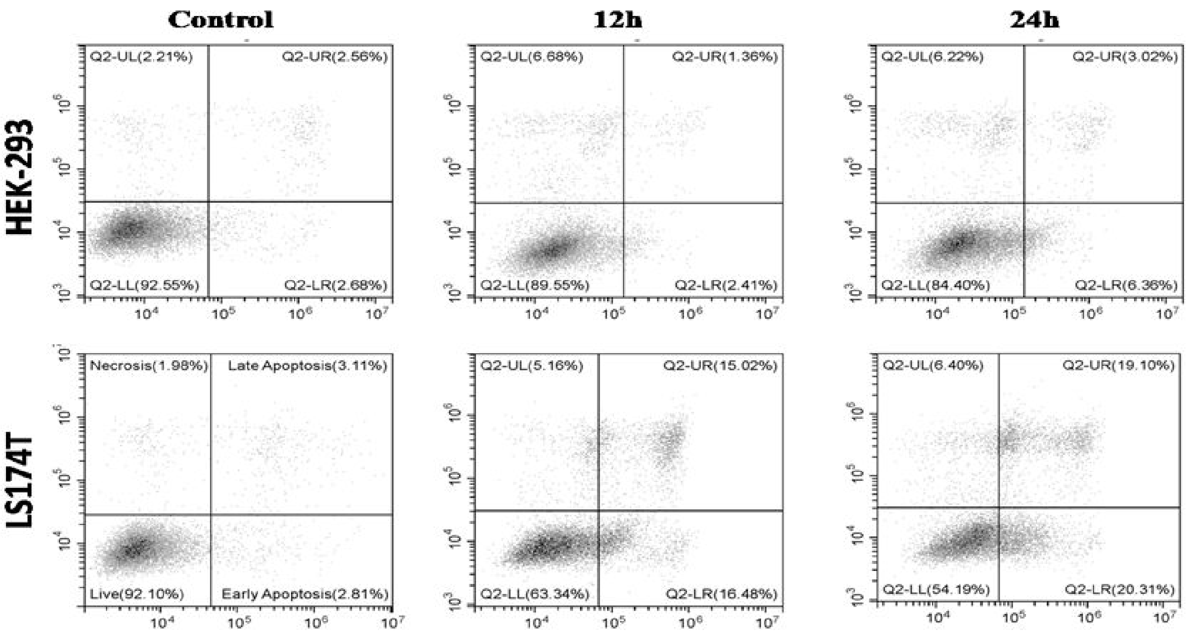

Figure S12: FACS analysis of 40 μg/ml Cbs-Cu-Af showed apoptosis in LS174T but no significant effect on HEK-293 cells. From 12h data it was observed that 63% of cells were live which further reduced to 54% at 24h in LS174T cells. HEK-293 cell survival was almost 84%. Cell death was mainly due to apoptosis. This indicates specific toxicity of Cbs-Cu-Af towards CEA expressing LS174T cells.

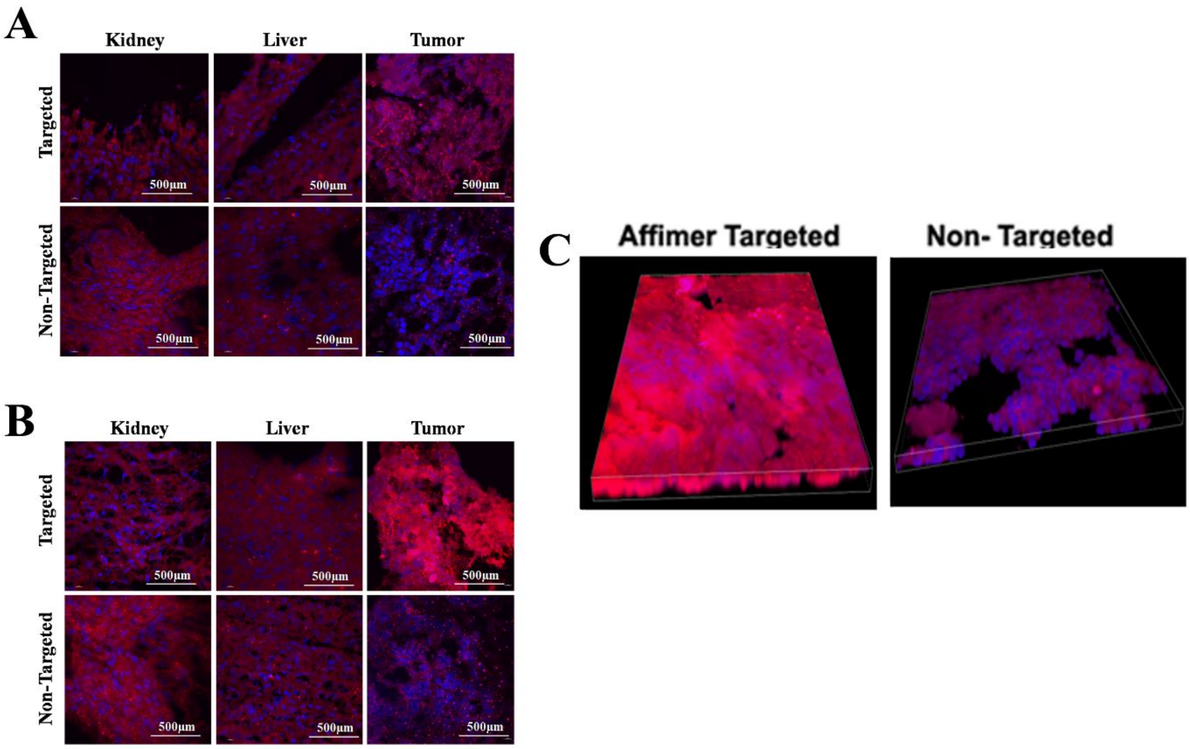

Figure S13: 5 μm tissue sections of kidney, liver tumor upon administration of Cbs-Cy5 (Non-targeted) and Cbs-Cy5-Af (Affimer targeted) analysed by confocal microscopy for Cy5 uptake after **(A)** 24h and **(B)** 48h. Cy5 uptake is maximum in the tumor compared to other organs after 48h; **(C)** 3D reconstruction of z-stack of tumor tissue section after 74h post administration of Cbs-Cy5-Af and Cbs-Cy5 showing an increased accumulation of Cy5 in the Affimer targeted group compared to the non-targeted.

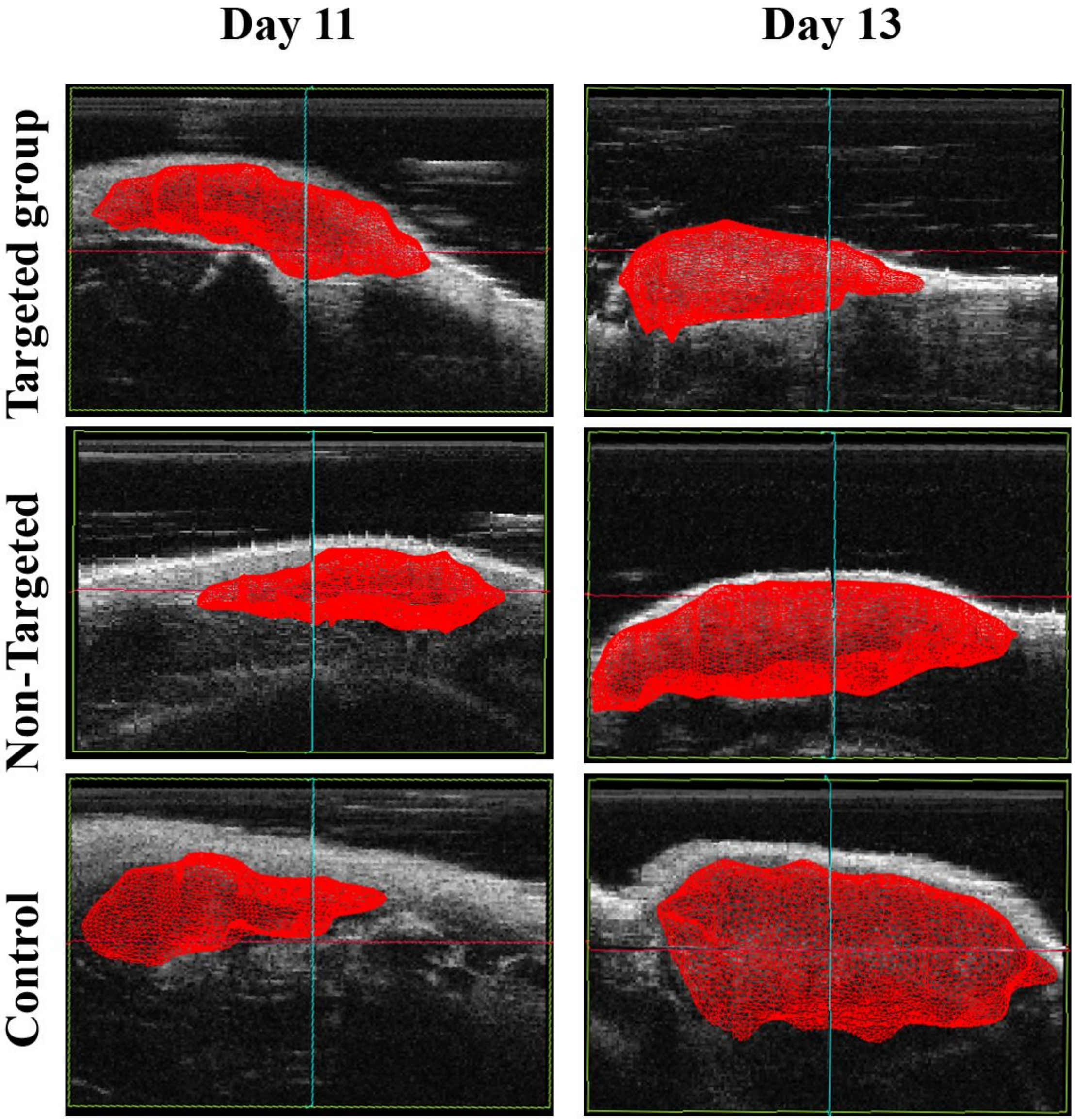

Figure S14: 3D reconstruction of tumor volume from ultra sonography measuredat day 11 and day 13 in the three groups of mice. The Cbs-Cu-Af (Affimer targeted) administered group showed restricted tumor growth on day 13 compared to the rest of the two groups.

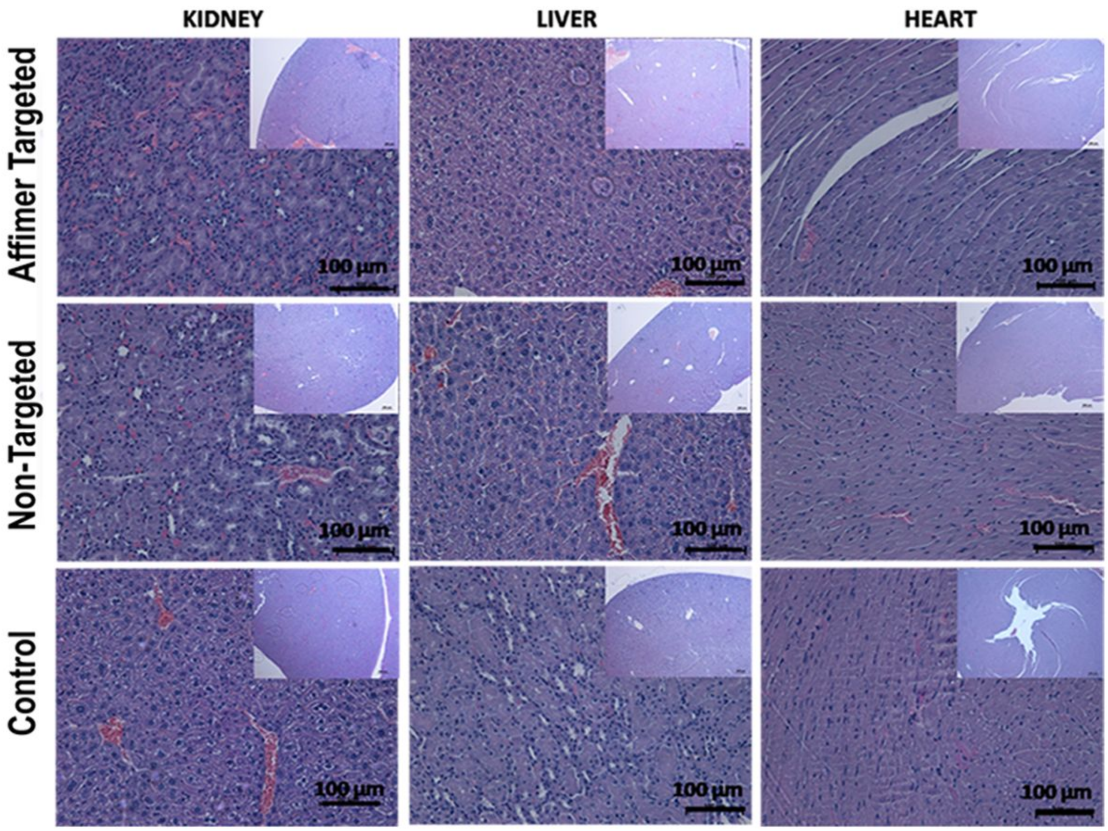

Figure S15: Off-target toxicity study in the three groups of mice by haematoxylin and eosin staining (H&E) of kidney, liver and heart. As evident from the images there is no sign of toxicity or tissue necrosis arising from administration of Cbs-Cu (non-targeted) or Cbs-Cu-Af (Affimer targeted) cubosomes.

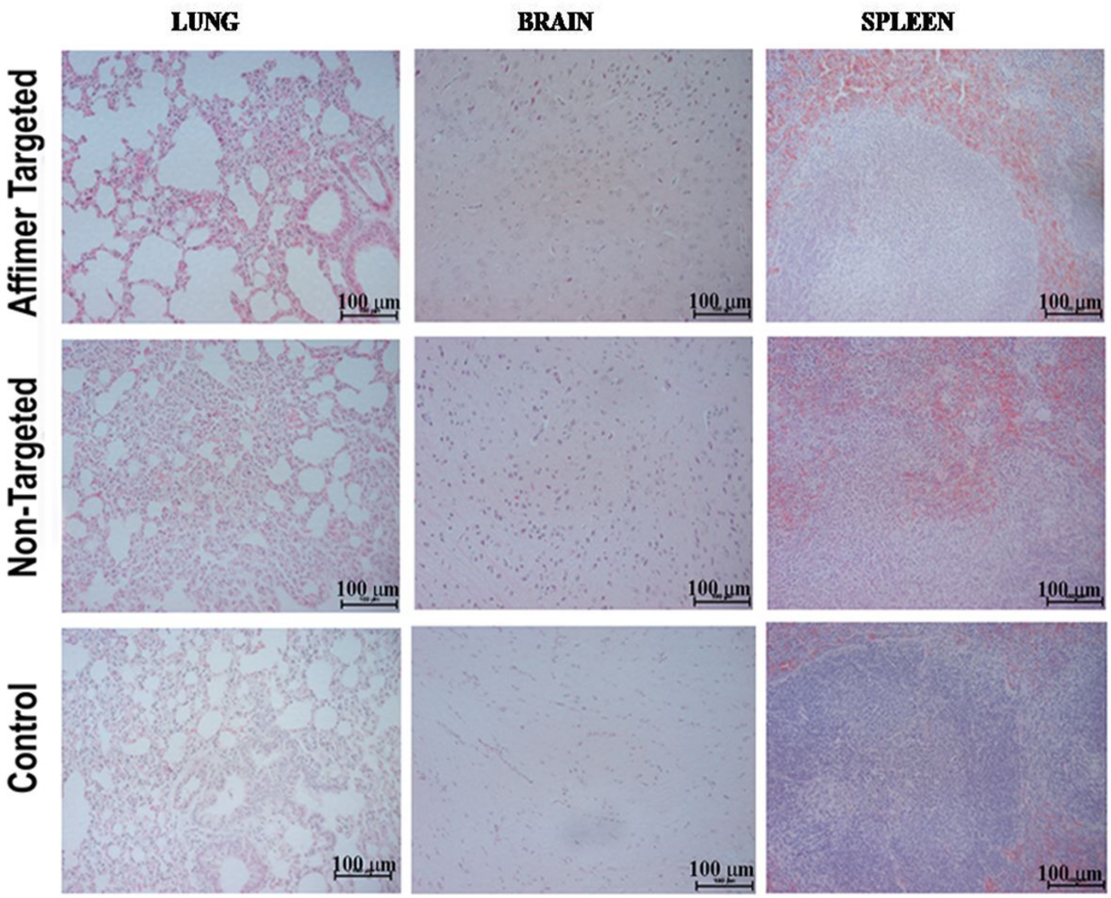

Figure S16: Off-target toxicity study in the three groups of mice by tissue examination of lung, brain and spleen by haematoxylin and eosin staining (H&E). As evident from the images there is no sign of toxicity or tissue necrosis arising from administration of Cbs-Cu (non-targeted) or Cbs-Cu-Af (Affimer targeted) cubosomes.

References

(1) Solomon, D.; Gupta, N.; Mulla, N. S.; Shukla, S.; Guerrero, Y. A.; Gupta, V. Role of In Vitro Release Methods in Liposomal Formulation Development: Challenges and Regulatory Perspective. *The AAPS Journal* **2017,** *19* (6), 1669-1681, DOI: 10.1208/s12248-017-0142-0.

(2) Nguyen, T. T. T. N.; Østergaard, J.; Stürup, S.; Gammelgaard, B. Determination of platinum drug release and liposome stability in human plasma by CE-ICP-MS. *International Journal of Pharmaceutics* **2013,** *449* (1), 95-102, DOI: https://doi.org/10.1016/j.ijpharm.2013.03.055.

(3) Shamsuddin, S. H.; Jayne, D. G.; Tomlinson, D. C.; McPherson, M. J.; Millner, P. A. Selection and characterisation of Affimers specific for CEA recognition. *Scientific Reports* **2021,** *11* (1), 744, DOI: 10.1038/s41598-020-80354-6.
(4) Shamsuddin, S. H.; Gibson, T. D.; Tomlinson, D. C.; McPherson, M. J.; Jayne, D. G.; Millner, P. A. Reagentless Affimer- and antibody-based impedimetric biosensors for CEA-detection using a novel non-conducting polymer. *Biosensors and Bioelectronics* **2021,** *178*, 113013, DOI: https://doi.org/10.1016/j.bios.2021.113013.
